# Neoantigen-reactive CD8^+^ T cells affect clinical outcome of adoptive cell therapy with tumor-infiltrating lymphocytes in melanoma

**DOI:** 10.1172/JCI150535

**Published:** 2022-01-18

**Authors:** Nikolaj Pagh Kristensen, Christina Heeke, Siri A. Tvingsholm, Annie Borch, Arianna Draghi, Michael D. Crowther, Ibel Carri, Kamilla K. Munk, Jeppe Sejerø Holm, Anne-Mette Bjerregaard, Amalie Kai Bentzen, Andrea M. Marquard, Zoltan Szallasi, Nicholas McGranahan, Rikke Andersen, Morten Nielsen, Göran B. Jönsson, Marco Donia, Inge Marie Svane, Sine Reker Hadrup

**Affiliations:** 1 Section for Experimental and Translational Immunology, Department of Health Technology, Technical University of Denmark (DTU), Kongens Lyngby, Denmark.; 2 National Center for Cancer Immune Therapy (CCIT-DK), Department of Oncology, Copenhagen University Hospital, Herlev, Denmark.; 3 Instituto de Investigaciones Biotecnológicas, Universidad Nacional de San Martín, Buenos Aires, Argentina.; 4 Danish Cancer Society Research Center, Copenhagen, Denmark.; 5 Cancer Genome Evolution Research Group, University College London Cancer Institute, London, United Kingdom.; 6 Section for Bioinformatics, Department of Health Technology, DTU, Kongens Lyngby, Denmark.; 7 Division of Oncology and Pathology, Department of Clinical Sciences Lund, Faculty of Medicine, Lund University, Lund, Sweden.

**Keywords:** Immunology, Therapeutics, Cancer immunotherapy, Melanoma, T cells

## Abstract

**BACKGROUND:**

Neoantigen-driven recognition and T cell–mediated killing contribute to tumor clearance following adoptive cell therapy (ACT) with tumor-infiltrating lymphocytes (TILs). Yet how diversity, frequency, and persistence of expanded neoepitope-specific CD8^+^ T cells derived from TIL infusion products affect patient outcome is not fully determined.

**METHODS:**

Using barcoded pMHC multimers, we provide a comprehensive mapping of CD8^+^ T cells recognizing neoepitopes in TIL infusion products and blood samples from 26 metastatic melanoma patients who received ACT.

**RESULTS:**

We identified 106 neoepitopes within TIL infusion products corresponding to 1.8% of all predicted neoepitopes. We observed neoepitope-specific recognition to be virtually devoid in TIL infusion products given to patients with progressive disease outcome. Moreover, we found that the frequency of neoepitope-specific CD8^+^ T cells in TIL infusion products correlated with increased survival and that neoepitope-specific CD8^+^ T cells shared with the infusion product in posttreatment blood samples were unique to responders of TIL-ACT. Finally, we found that a transcriptional signature for lymphocyte activity within the tumor microenvironment was associated with a higher frequency of neoepitope-specific CD8^+^ T cells in the infusion product.

**CONCLUSIONS:**

These data support previous case studies of neoepitope-specific CD8^+^ T cells in melanoma and indicate that successful TIL-ACT is associated with an expansion of neoepitope-specific CD8^+^ T cells.

**FUNDING:**

NEYE Foundation; European Research Council; Lundbeck Foundation Fellowship; Carlsberg Foundation.

## Introduction

Adoptive cell therapy with expanded tumor-infiltrating lymphocytes (TIL-ACT) can mediate durable tumor regression in patients with metastatic melanoma (1, 2). Furthermore, TIL-ACT has a high objective response rate even after the failure of checkpoint inhibitor therapy (1–4). TIL-ACT therefore represents an attractive treatment option for metastatic melanoma patients with high unmet medical needs. Current predictors of tumor regression and long-term survival after ACT include tumor-mutational burden (TMB) and neoantigen load (5), which have recently emerged as independent predictors of outcome across multiple immunotherapies (6, 7). Moreover, transcriptomic evidence implicates antigen presentation within the tumor microenvironment before TIL-ACT (5) as an important additional factor, suggesting that antigen presentation and immune recognition of mutation-derived neoantigens contribute to therapeutic benefit in TIL-ACT. While immune recognition and tumor cell killing are generally associated with a positive outcome (8, 9), evaluation of T cell recognition of mutation-derived neoantigens within TIL infusion (TIL Inf) products and peripheral blood after infusion have only been reported in case studies of complete responders (CRs) (10–15). We aimed to systematically assess T cell recognition toward neoantigens in TIL-ACT and the influence of such recognition on therapeutic outcome. Recent advances in T cell technologies have led to the possibility of comprehensive screening of T cell recognition against large libraries of patient-derived neoepitopes (12, 16–18). Here, we used DNA barcode–labeled pMHC multimers to screen for CD8^+^ T cell recognition, using 151 to 585 predicted neoepitopes per patient, presented in a multimeric form in the context of patient-matched HLA-I molecules (19, 20). Using this strategy, we determined the presence of CD8^+^ T cells recognizing mutation-derived neoepitopes, here denoted as neoantigen-reactive T cells (NARTs), in TIL Inf products from 26 patients with metastatic melanoma. Furthermore, we examined the persistence of such T cells in samples of peripheral blood collected at multiple time points after therapy. This comprehensive mapping of NARTs demonstrates a substantial T cell reactivity level toward patient-derived neoepitopes and a positive influence on clinical outcome following TIL-ACT. This highlights the importance of detecting and enhancing the levels of such T cells in TIL-ACT.

Moreover, this study provides essential data to support efforts to identify the few immunogenic neoepitopes that give rise to T cell recognition out of the large number of predicted neopeptides. Recent efforts have been made to identify the parameters that determine the immunogenicity of a given neoepitope (21) and facilitate more accurate prediction of such sequences for therapeutic measures. In the current study, we evaluated a total of 5921 predicted neopeptides and identified T cell recognition toward 106 (1.8 %) of these in TIL Inf products. Using this large data set, we further assessed the influence of HLA binding, antigen expression level, clonality, TMB, and type of mutation on immunogenicity (i.e., recognition of a given neopeptide).

## Results

### Identification of neoepitope-reactive CD8^+^ T cells.

In a cohort of metastatic melanoma patients treated with TIL-ACT (Supplemental Table 1; supplemental material available online with this article; https://doi.org/10.1172/JCI150535DS1), prediction of patient-specific mutated HLA-I epitopes was performed using whole exome sequencing (WES) and RNA-Seq on tumor material and normal tissue PBMCs. The in silico neopeptide prediction platform MuPeXI (https://services.healthtech.dtu.dk/service.php?MuPeXI-1.1) was employed to identify single nucleotide variants and indels/frameshifts from the sequencing data specific to the cancer material (5, 20). Mutation-derived peptides were subsequently ranked using netMHCpan (20, 22) and transcription of the corresponding gene (transcripts per million [TPM]) (see Methods) with the aim of including at least 200 neopeptides per patient.

We covered 30 different HLA alleles ranging from 2 to 6 HLA alleles per patient (average, 4.4 HLAs) (Supplemental Figure 1, A and C); however, HLA-C*02:02 and C*05:01 were excluded from data analyses due to technical concerns. Thus, the final neopeptide library ranged from 151 to 585 peptides per patient (Supplemental Figure 1B), with the most frequent alleles in our cohort being HLA-A*01:01 and C*03:04 (Supplemental Figure 1C). In addition to neopeptides, we also included a small set of known CD8^+^ T cell epitopes derived from common human viruses EBV, CMV, and influenza virus (FLU). These represent “bystander” T cells in the TIL Inf product and also serve as positive controls for the technical process.

**Figure 1:**
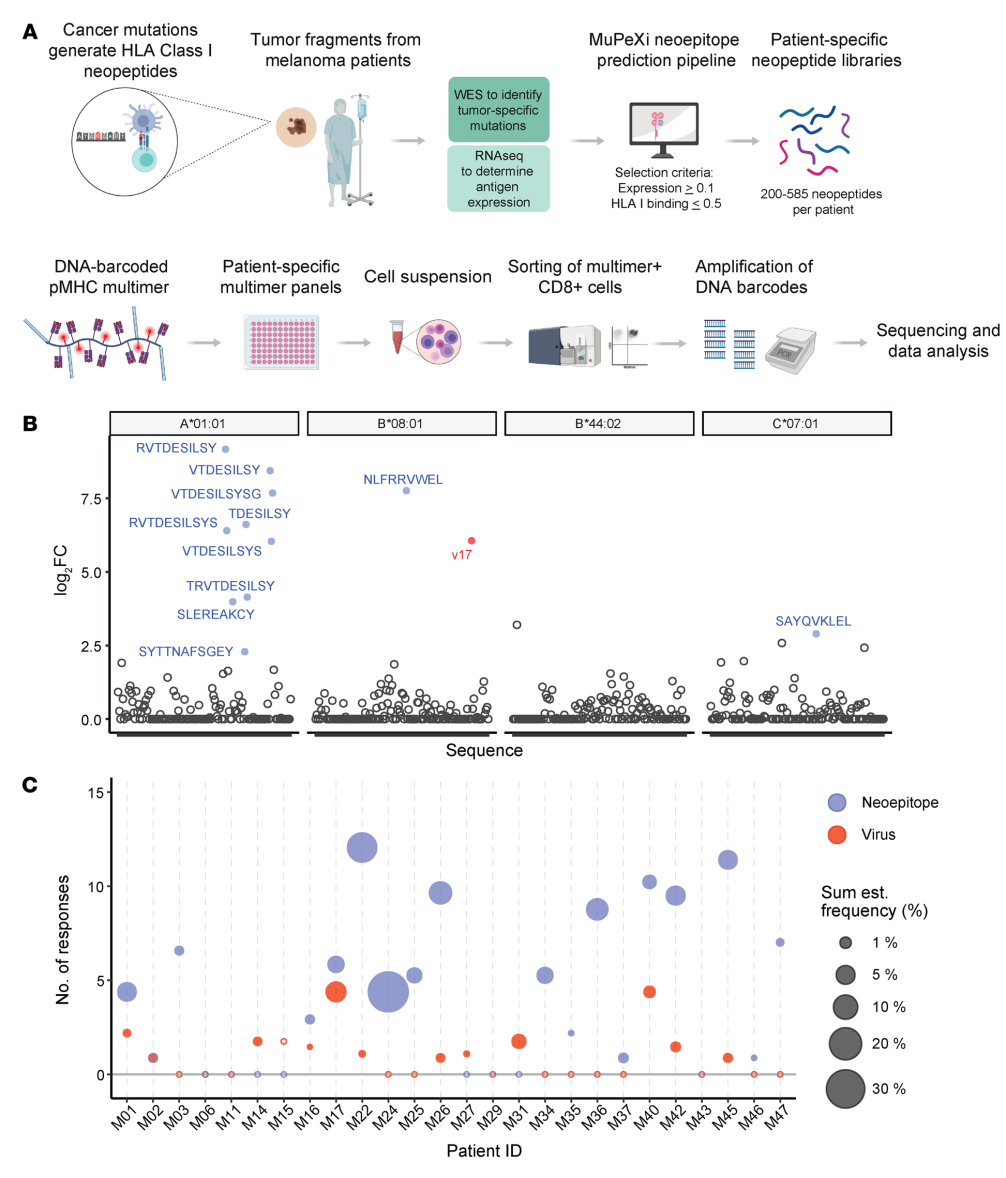
Detection of neoepitope-specific CD8^+^ T cells in expanded TILs of melanoma. (**A**) Melanoma-specific mutation-derived peptides were predicted to bind patient’s HLA molecules using the prediction platform MuPeXI. DNA barcode–labeled MHC multimers with either neopeptides or virus-derived peptides were assembled on a PE-labeled streptavidin-conjugated dextran backbone. Multimer-binding NARTs were fluorescence sorted and T cell specificities decoded by barcode sequencing. (**B**) Examples of neoepitope- and virus-specific CD8^+^ T cells detected in expanded TILs of melanoma patient M22 (PR) across available HLAs. Significant barcode enrichment is defined based on a log_2_ FC of the number of barcode reads compared with triplicate baseline samples. *P* ≤ 0.001 (egdeR) after correction for multiple hypothesis testing (see Methods). Blue, NARTs; red, virus-specific CD8^+^ T cells; black, multimers with nonenriched barcodes. V17 annotate EBV peptide RAKFKQLL. (**C**) Number and frequency of neoepitope- and virus-specific CD8^+^ T cells in TIL samples across cohort of 26 melanoma patients. Blue, NARTs; red, virus-specific CD8^+^ T cells. Number of and frequency of NARTs were normalized to absolute HLA coverage (see Methods). Sum est. frequency, sum of estimate frequency.

DNA barcode–labeled neopeptide libraries were constructed as described previously (19) using UV-mediated peptide-MHC exchange (23, 24) and fluorescent streptavidin-labeled dextrans (see Methods). PBMCs and TIL Inf products were stained with patient-specific multimer libraries followed by sorting of multimer-binding CD8^+^ T cells. The coattached DNA barcodes were amplified from the sorted T cell population to reveal antigen specificity (ref. 19 and Figure 1A). We defined biologically relevant NARTs as NARTs with an estimated frequency of at least 0.01% and without presence in partially HLA-matching healthy donor PBMCs. To assess the reproducibility of our pMHC multimer library screens, we screened TIL samples of 9 patients twice with the same library, demonstrating a correlation between technical replicates (*R* = 0.55; *P* < 2.2 × 10^–10^; Supplemental Figure 1D).

An example of the analysis of enriched DNA barcodes and their corresponding pMHC in a TIL Inf product from patient M22 ( partial responder [PR]) is depicted in Figure 1B and for patients M14 (progressive disease [PD]) and M26 (CR) in Supplemental Figure 2. In patient M22, NARTs were detected for 3 of 4 HLA molecules included, although most reactivity was seen against HLA-A*01:01–restricted peptides. Of interest, 7 HLA-A*01:01–restricted neoepitopes recognized by the M22 TIL Inf product comprised the C-terminal amino acid sequence SILSY (AKAP9^P1796L^), and CD8^+^ T cells specific for each of these peptides were confirmed in TIL Inf products with single-tetramer staining (Supplemental Figure 3A).

From in silico structural models of the interaction between the different AKAP9^P1796L^ peptide variants and the HLA-A*01:01 molecule, we observed that leucine (L), introduced by the mutation, protruded from the HLA-binding groove for potential interaction with a TCR. Furthermore, the four 8 to 10 mer epitope variants shared this conformation when bound to HLA-A*01:01 (Supplemental Figure 3B). This suggests that the AKAP9^P1796L^ amino acid substitution has given rise to multiple neoepitopes that may be recognized by the same population of CD8^+^ T cells, but with different affinities. The binding affinity hierarchy can be assessed both by the estimated frequency (Supplemental Figure 3C) and the MFI of the tetramer populations (Supplemental Figure 3D) and indicates favorable interaction with the 9 mer and 10 mer neoepitopes holding the SILSY motif.

Screening of TIL Inf products from 26 melanoma patients with personalized multimer libraries resulted in the detection of 106 different NART populations across the cohort. NARTs were detected in 18 out of 26 TIL Inf products, ranging from 0 to 13 NART populations per sample. To avoid any potential bias based on differences in HLA coverage, the number and frequency of detected NARTs were normalized to the average HLA coverage of the cohort (4.4 HLAs per patient). Following HLA normalization, the median number of NARTs per TIL Inf product was 3.7 (range 0–12.1, Figure 1C). Additionally, we detected the presence of virus-specific CD8^+^ T cells toward a selected list of virus-derived epitopes in half of the TIL Inf products (13 out of 26 patients, Figure 1C), which is in line with previous analyses of TIL Inf products (25, 26). Across all TIL Inf products, we observed an estimated NART frequency of 0%–38.6% (median = 0.63%) of total CD8^+^ T cells (Figure 1C).

### Recognition of melanoma tumor cells by NARTs in vitro.

**Figure 2:**
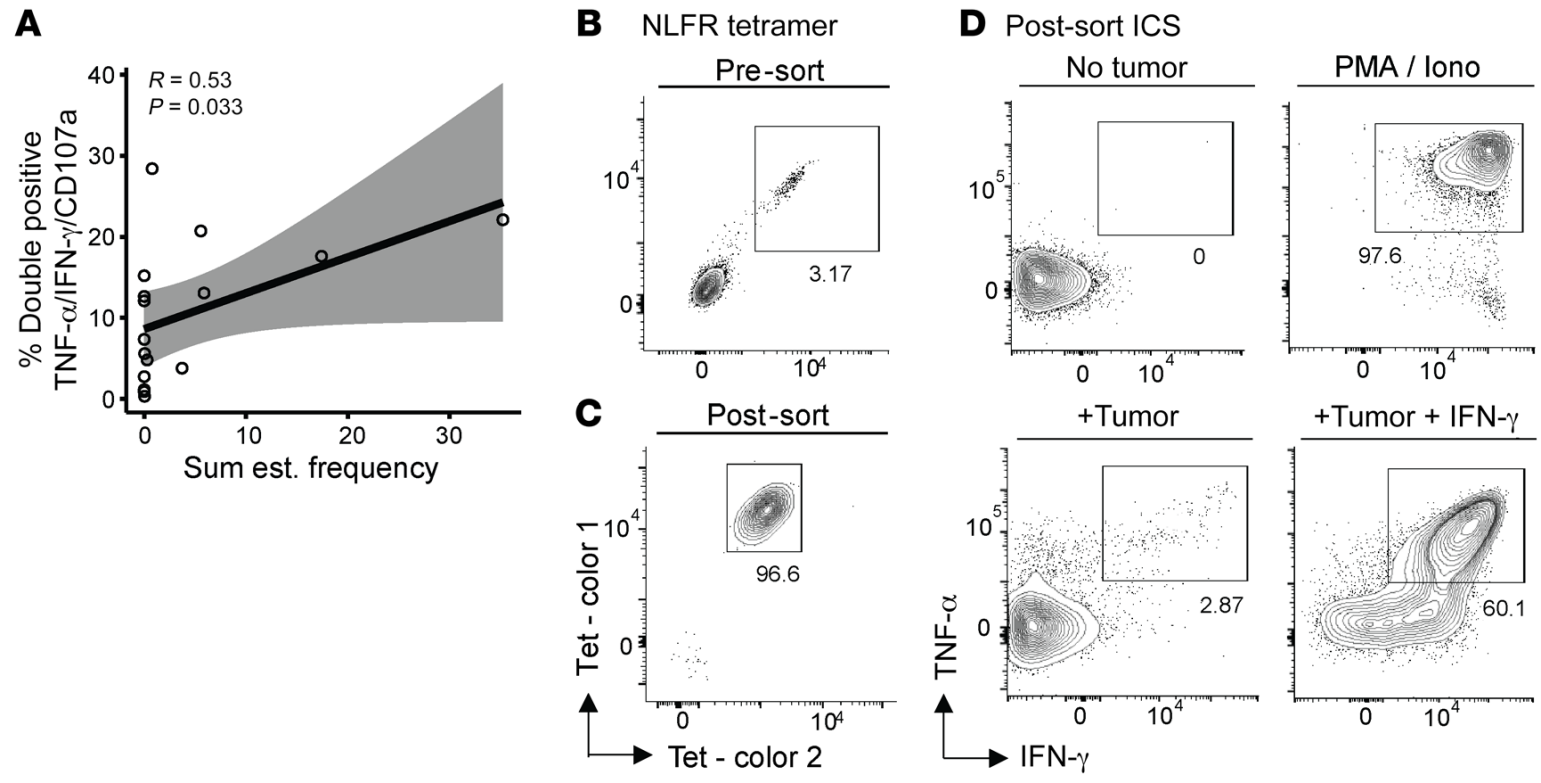
Autologous tumor recognition by enriched NARTs. (**A**) Correlation of TIL reactivity to autologous tumor (measured by intracellular cytokine staining) and sum of estimated NART frequency. TIL reactivity toward an autologous tumor cell line was defined as positive for 2 out of the 3 proteins TNF-α, IFN-γ, and CD107a. Sixteen patients with available tumor reactivity data were included from both responder (*n* = 6) and nonresponders (*n* = 10). *R* and *P* values from Spearman’s correlation with 95% CIs in gray. NART frequency was normalized to absolute HLA coverage (see Methods). (**B** and **C**) HLA-B*08:01–restricted, NLFR-specific CD8^+^ T cells from M22 TIL Inf product were sorted based on 2-color tetramer binding (**B**) and expanded in vitro followed by NLFR-tetramer staining (**C**). (**D**) Tumor reactivity as measured by TNF-α/IFN-γ release after coculture of expanded, NART-specific cell products with or without autologous tumor cell lines, with PMA/ionomycin or with autologous tumor cell line and IFN-γ. NLFR, NLFRRVWEL from USP34^S1391F^. TIL reactivity data shown in **A** originate from previous study (4), and the assay was performed as described previously (66).

The TIL Inf product from most patients (16 of 26) was previously analyzed for tumor recognition properties in terms of cytokine secretion toward an autologous tumor cell line, generated from the same tumor biopsy as the TIL Inf product (4). The estimated frequency of NARTs identified in this study correlated with the capacity of the TIL Inf product to recognize the tumor, indicating that detected NARTs may indeed contribute to tumor cell recognition (Figure 2A). While a significant association was observed, the effect on cytokine secretion from other immune subsets, tumor antigen classes, or NARTs restricted to HLA alleles not included in our study cannot be excluded.

We additionally investigated the direct tumor-recognition capacity of sorted and expanded neoepitope-specific T cell populations. From the patient M22 TIL Inf product, we sorted USP34^S1391F^–derived NLFR-HLA-B*08:01–specific T cells using tetramers. The presence of such T cells was verified (3.2%, Figure 2B), and postsort expansion resulted in purity of greater than 96% (Figure 2C). The expanded NLFR-HLA-B*08:01–specific T cells displayed tumor recognition determined by cytokine secretion upon coculture with an autologous tumor cell line with (60.1%) and without (2.87%) pretreatment with IFN-γ (Figure 2D). Thus, tumor recognition was specific and greatly enhanced by IFN-γ pretreatment of the autologous tumor cell line. It has previously been demonstrated that IFN-γ pretreatment enhances MHC-I expression and antigen presentation in both autologous (8) and established tumor cell lines (27). We also sorted CD8^+^ T cells specific to 2 AKAP9^P1796L^ peptide variants followed by rapid expansion (Supplemental Figure 4A), which recognized their respective AKAP9^P1796L^ variants (Supplemental Figure 4B). These sorted T cell populations both recognized autologous tumor cell lines with and without pretreatment with IFN-γ (Supplemental Figure 4C). This indicates that some multimer-detected NARTs are capable of further expansion and can specifically recognize autologous tumor cell lines.

### The number and frequency of NARTs are associated with the clinical outcome of TIL-ACT.

**Figure 3:**
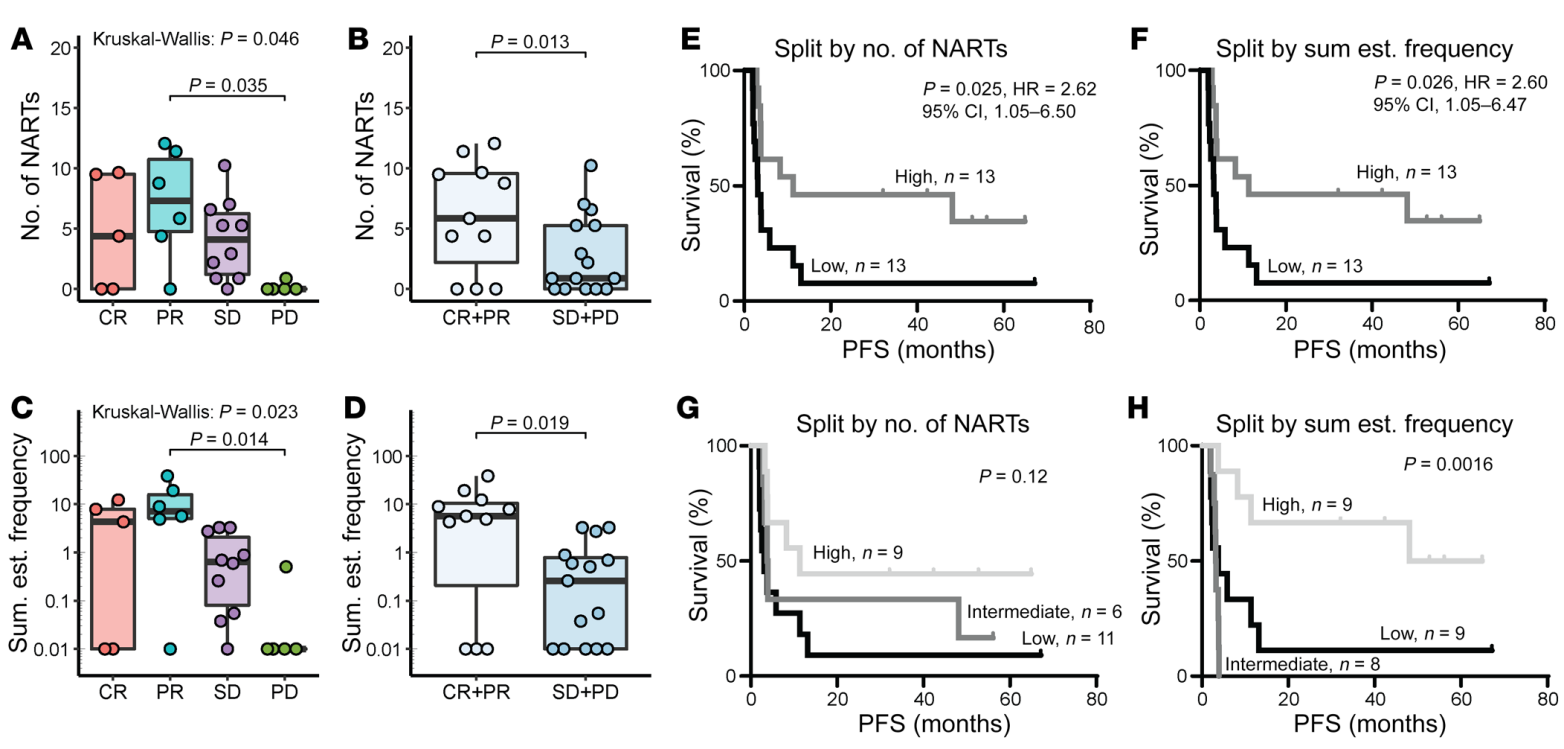
Frequency of NARTs correlates with increased survival after TIL-ACT. (**A** and **B**) NART diversity represented as the number of NARTs detected in TIL Inf products for each patient according to RECIST (**A**) and clinical response (**B**). (**C** and **D**) NART frequency represented as the sum of estimated frequency of NARTs detected in TIL Inf products for each patient according to RECIST (**C**) and clinical response (**D**). (**E** and **F**) PFS for the cohort split by median NART diversity (median = 3.65 NARTs) (**E**) and median NART frequency (median = 0.63 %) (**F**). (**G** and **H**) PFS for the cohort splits by high (>66th percentile), intermediate (> 33rd percentile), and low groups (≤33rd percentile). (**G**) NART diversity. 66th percentile = 5.65 NARTs. 33rd percentile = 0.88 NARTs. (**H**) NART frequency. 66th percentile = 3.26%. 33rd percentile = 0.03%. *P* values were calculated using Kruskal-Wallis test followed by Dunn’s multiple comparison test in **A** and **C**; only significant comparisons are shown. Nonparametric Mann-Whitney *U* test was used for **B** and **D**. Box plot whiskers represent IQR. *P* values and HRs were calculated using the Mantel-Cox test and log-rank approach, respectively (**F**). *P* values for **G** and **H** were calculated using log-rank test. Both number of and frequency of NARTs were normalized to absolute HLA coverage (see Methods). *n* = 26 for all plots. All values displayed on a logarithmic scales were increased by 0.01 to account for 0 values.

Next, we investigated whether higher diversity (number of responses) and frequency of NARTs in TIL Inf products correlate with improved clinical efficacy of TIL-ACT. NARTs were detectable across all Response Evaluation Criteria in Solid Tumors (RECIST), version 1, groups (28), although they were severely depleted from TIL Inf products given to patients that developed PD (*n* = 6) (Figure 3A). Overall, NARTs tended to demonstrate greater diversity in products from responders compared with nonresponders (Figure 3B).

The estimated NART frequency within TIL Inf products was significantly higher in responders compared with nonresponders (Figure 3, C and D, and Supplemental Figure 5, A and B), suggesting that NART frequency affects clinical outcome. Tumor mutational burden and number of predicted neoepitopes were uniformly distributed across RECIST groups (Supplemental Figure 5, D and H), and no difference was observed between responders and nonresponders (Supplemental Figure 5, E and I). Tumor mutational burden was, however, associated with longer progression-free survival (PFS) (Supplemental Figure 5F), as previously indicated (5), although we did not observe a strong influence of the number of predicted neoepitopes on PFS (Supplemental Figure 5J).

Next, we investigated whether the diversity and frequency of NARTs within TIL Inf products affected PFS and overall survival (OS). Patients in whom the number of NARTs was above the median of 3.7 (high, *n* = 13) had increased PFS (*P* = 0.025, HR 2.62; 95% CI = 1.05–6.50) compared with patients below the median (low, *n* = 13; Figure 3E). Likewise, patients with a high NART frequency within TIL Inf products (median = 0.7%) (high, *n* = 13) demonstrated significantly improved PFS (*P* = 0.026, HR 2.60; 95% CI = 1.05–6.47) compared with patients with low NART frequency (low, *n* = 13; Figure 3F). High NART frequency also showed a positive effect on OS (Supplemental Figure 6B); however, no such correlation was found with NART diversity (Supplemental Figure 6A). Note that OS might also be affected by subsequent treatment given after TIL-ACT.

Interestingly, the clinical impact of NART frequency was most prominent for patients above the 66th percentile. For NART frequency, the high patient group (above the 66th percentile, *n* = 9) showed significantly longer PFS (*P* = 0.0016; Figure 3H) and OS (*P* = 0.021; Supplemental Figure 6D) compared with the intermediate patients (equal to or below the 66th percentile and greater than the 33rd percentile, *n* = 8) or low patients (equal to or below the 33rd percentile, *n* = 9). In contrast, NART diversity did not significantly affect survival (PFS and OS) when comparing groups split by the 66th and 33rd percentiles (Figure 3G and Supplemental Figure 6C). The 66th and 33rd percentiles corresponded to a frequency of 3.26% and 0.03%, respectively, while the same percentiles for NART diversity were 5.65 and 0.88 NARTs, respectively.

In our analysis, T cells that recognized different overlapping peptides originating from the same mutation were defined as multiple individual NART populations. However, T cell recognition of multiple neopeptides could also arise from crossreactivity of a single NART population toward several similar epitopes. To avoid any bias in our data analyses based on such potential crossreactive T cell populations, we reduced the number of detected NART responses to the number of unique immunogenic somatic mutations recognized by NARTs (median = 2.6) and redid our survival analysis using the most frequent NART as a proxy for recognition of all overlapping epitopes from the same nonsynonymous mutation. The result showed a similar association: both NART diversity and frequency correlated with increased PFS, whereas only frequency correlated with increased OS (Supplemental Figure 6, E–H), ensuring that contribution from T cell recognition of overlapping epitopes did not bias our overall observation. In summary, these data suggest that high frequency of NARTs positively affects therapeutic outcome following TIL-ACT.

### NARTs are detected in peripheral blood after TIL-ACT and decline over time.

**Figure 4:**
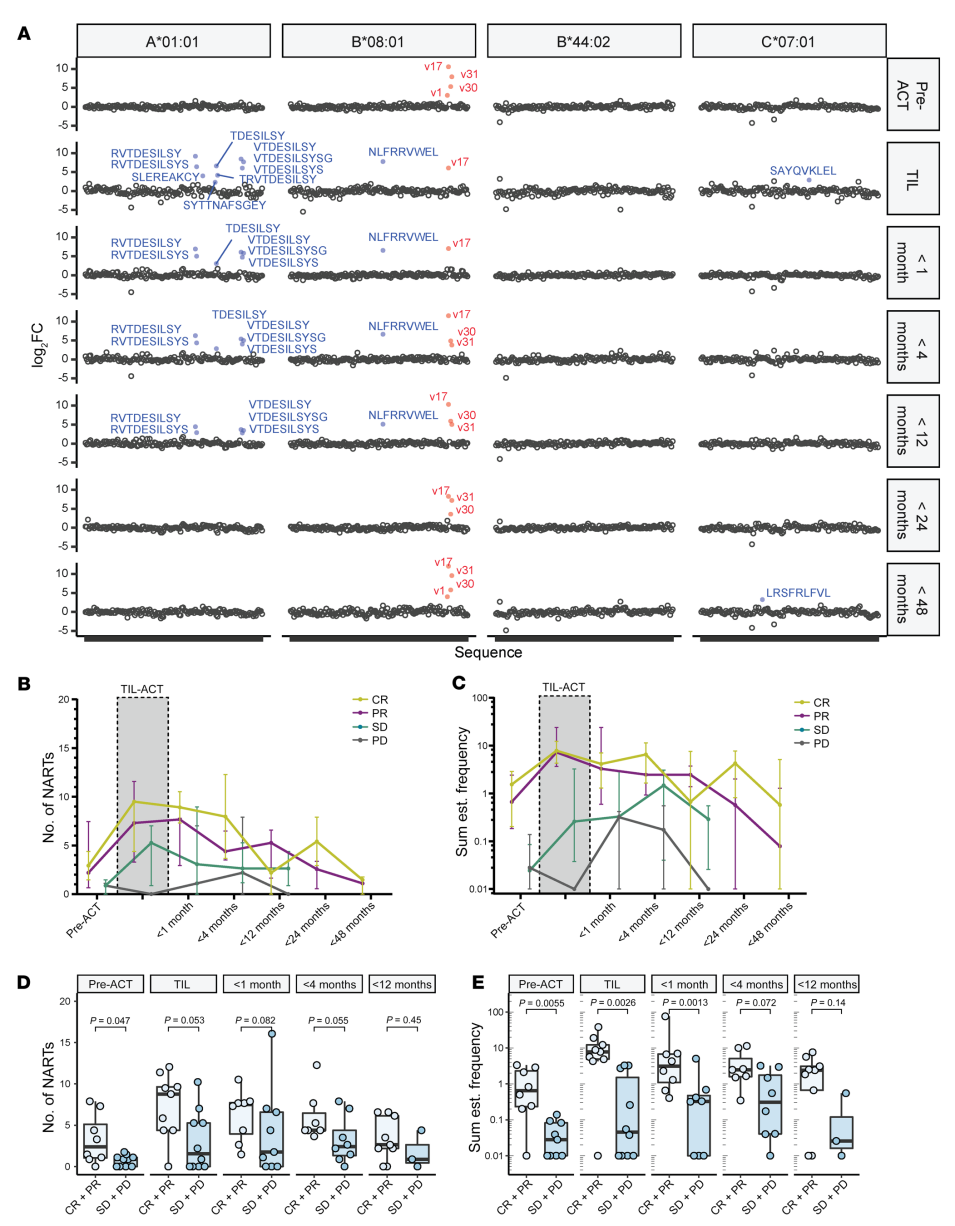
NARTs appear in peripheral blood and decline in frequency following TIL-ACT. (**A**) Output example from screening paired PBMCs from 19 patients. Virus- and neoepitope-specific CD8^+^ T cells in patient M22 (PR) in pre-ACT PBMCs, TIL Inf product, and PBMCs following TIL-ACT. Blue, NARTs; red, virus-specific CD8^+^ T cells; black, multimers associated with nonenriched barcodes. Significant barcode enrichment is defined based on a log_2_ FC of the number of barcode reads compared with triplicate baseline samples. *P* < 0.001 (egdeR) (see Methods). V1 annotate FLU peptide ELRSRYWAI, v17 annotate EBV peptide RAKFKQLL, v30 annotate EBV peptide QAKWRLQTL, and v31 annotated EBV peptide FLRGRAYGL. (**B** and **C**) Median number of NARTs. Error bars indicate IQR. Points were displaced for visual purposes. (**B**) Number of NART responses and sum of estimated NART frequency (**C**) over time in TIL Inf product and available PBMC samples. Patients were divided according to RECIST groups. (**D** and **E**) Box plots representing diversity (**D**) and frequency (**E**) of NARTs for each patient according to RECIST groups. *P* values were calculated using Mann-Whitney *U* test. Nineteen patients had both TIL Inf products and PBMCs available, but the number of samples at each time point varied according to sample and data availability (Supplemental Table 1 and Supplemental Figure 8). NART frequency could not be calculated for M40 PBMCs before ACT and for M40 PBMCs less than 1 month after treatment (see Methods) and are therefore excluded in **C** and **E**. Whiskers represent IQR. NART frequencies were normalized to HLA coverage of the given patient (see Methods). All values displayed on logarithmic scales were increased by 0.01 to account for 0 values.

As indicated by others (10, 29), an essential factor for TIL-ACT efficacy is the capacity of transferred T cells to persist in patients following therapy. This can be measured based on their presence in peripheral blood over time after transfer. For 19 patients, available blood samples were taken 8 days before TIL-ACT and at different time points after TIL Inf, i.e., less than 1 month after TIL-ACT, less than 4 months after TIL-ACT, less than 12 months after TIL-ACT, less than 24 months after TIL-ACT, and less than 48 months after TIL-ACT (Supplemental Table 1). NARTs present in the first or later PBMC samples after ACT were defined as engrafted. Furthermore, if a given NART was detected in multiple later PBMC samples, that NART was regarded as persisting. Each sample was screened for T cell recognition toward neopeptides included in the full patient-specific neoepitope-MHC library, exemplified by patient M22 (PR) (Figure 4A). In M22, only virus-specific T cells could be detected in the pre-ACT PBMC sample, namely, B*08:01-restricted CD8+ cells capable of binding epitopes FLU-ELR (v1), EBV-RAK (v17), EBV-QAK (v30), and EBV-FLR (v31). These virus-specific CD8^+^ T cells were detectable throughout most time points, while NARTs engrafted (PBMC <1 month) and persisted in the following PBMC samples up to 1 year after treatment.

Similar NART kinetics were observed in patient M45 (PR), with NARTs recognizing overlapping neoepitope containing the mutated sequence SAGA (SORC2^A1093S^) (Supplemental Figure 7). SORC2^A1093S^ was first recognized in the M45 TIL Inf product, and immune recognition persisted in PBMCs until the last recorded time point (<12 months). Furthermore, M45 showed immune recognition toward the same neoepitope DIHF (ZNF786^M87I^) bound to multiple HLA alleles (HLA-A*01:01, A*24:02, and B*13:02). Recognition of ZNF786^M87I^ was initially discovered in the TIL Inf product, and while it persisted on HLA-A*24:02 until the last time point for M45, it appeared to incompletely persist on HLA-A*01:01 and B*13:02. Overall, this suggests that ZNF786^M87I^ produces a promiscuous neoepitope capable of binding multiple HLAs, with a preference for HLA-A*24:02. HLA promiscuity is otherwise known to occur for viral epitopes (30).

The median NART diversity and frequency across RECIST categories were followed to assess the overall kinetics of NARTs after ACT. Note that most nonresponders did not have PBMC samples for less than 12 months and thereafter (7 of 10). NART diversity increased markedly when comparing pre-ACT PBMCs and the TIL Inf product and declined over time after TIL-ACT in the CR, PR, and stable disease (SD) patient groups, displaying the expansion of NART populations in the TIL Inf product and their persistence after therapy (Figure 4B). NART frequency demonstrated kinetics similar to those of NART diversity. However, only responders appeared to have substantial frequencies of NARTs within TIL Inf products (Figure 4C). Unlike those in the other groups, patients with PD did not display any NARTs within TIL Inf products (*n* = 3); however, they did appear to have ongoing NART recognition in peripheral blood before and after therapy, although at lower frequencies (Figure 4, B and C).

Finally, we compared responders and nonresponders in relation to NART diversity across all time points and found that responders had a higher level of NART diversity in PBMCs collected before TIL-ACT (Figure 4D). Similarly, we found increased NART frequency in responders before TIL-ACT, within TIL Inf products, and at early time points following infusion (>1 month; Figure 4E).

In conclusion, we observed a broad repertoire of NARTs recognizing single neoepitopes, overlapping neoepitopes, and HLA promiscuous neoepitopes in TIL Inf products of metastatic melanoma patients treated with TIL-ACT. These NARTs showed signs of engraftment and could persist in peripheral blood after TIL-ACT. Furthermore, we observed that responders had a higher estimated NART frequency before and following TIL-ACT in peripheral blood, supporting prior prospective efforts (31).

### Engrafted neoepitope-specific CD8^+^ T cells dominate immune recognition in responders of TIL-ACT.

To better understand the dynamic relationship among preexisting, ongoing, and TIL-derived immune recognition, we annotated each detected NART according to its first appearance from 8 days prior to therapy (pre-ACT PBMCs) to the last available time point. Thus, if a NART population appeared exclusively in pre-ACT samples, it was annotated pre-ACT. If a given NART was detected in both pre-ACT PBMCs and in the given TIL Inf product, it was denoted pre/TIL, while if it first appeared in the infusion product, it was denoted TIL. Finally, if a NART population first appeared in a later PBMC sample it was regarded as novel, annotated with its first time of appearance and followed from there on out (see patient overview in Supplemental Figure 8).

**Figure 5:**
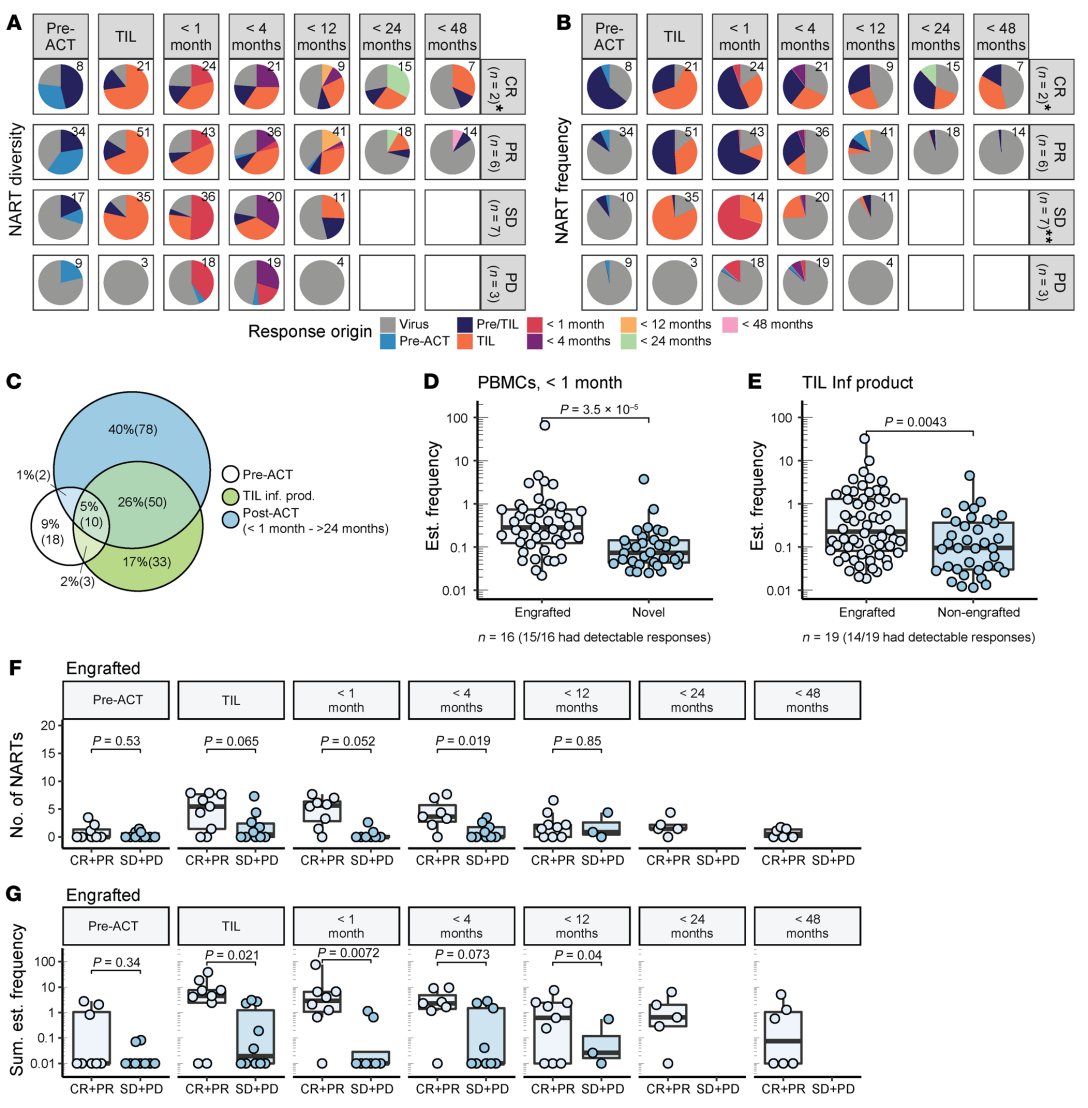
Responding patients have high-frequency engrafting NARTs in their TIL Inf product. (**A** and **B**) Each NART population was annotated and colored according to first appearance in pre-ACT PBMCs, TIL Inf products, and post-ACT PBMCs (<1 month to <48 months). Black numbers specify the total number of NARTs detected for the specific time and RECIST group. (**A**) Distribution of NARTs within RECIST groups according to first appearance. (**B**) Distribution of NART frequency within RECIST groups according to first appearance. *M01 (CR) did not have pre-ACT and <1 month PBMCs available and was excluded from analysis to avoid a biased distribution. **Frequency data could not be calculated for M40 pre-ACT and M40 post-ACT <1 month, which were excluded (see Methods). (**C**) Venn diagram showing the overlap of detected NARTs among pre-ACT PBMCs, TIL Inf products, and all post-ACT PBMC samples. *n* = 19. (**D**) The estimated frequency of each NART population detected less than 1 month after infusion. Responses were either regarded as engrafted (i.e., also detected in TIL Inf) or novel. *n* = 16. M01 and M40 were excluded as stated for **A** and **B**; M29 did not have detectable antigen-specific CD8^+^ T cells before the second time point after ACT. (**E**) The estimated frequency of each NART population observed in TIL Inf products. Nonengrafted versus engrafted (i.e., detected at least once at a later time points). *n* = 19. (**F** and **G**) Number and frequency of engrafted NARTs, defined by presence in both TIL Inf product and after TIL-ACT. *n* varied according to sample availability (Supplemental Table 1 and Supplemental Figure 8). M40 before ACT and <1 month PBMCs were excluded from **G** (see Methods). Sum of estimated frequency in **G** was increased by 0.01 to account for 0 values. *P* values from Mann-Whitney *U* test. Whiskers represent IQR.

Using this categorization, we observed that persisting NARTs derived from the TIL Inf product (Pre/TIL plus TIL) were present across responders and patients with SD at multiple time points after infusion, but absent in patients with PD (Figure 5, A and B, and Supplemental Figure 8). Additionally, we observed that 7 of 8 responders and 5 of 10 nonresponders with available pre-ACT material had preexisting NARTs (pre-ACT plus pre/TIL). Preexisting NARTs are likely clinically relevant, as TIL Inf products from responders were overall dominated by preexisting immune recognition that was further expanded to high frequencies within the TIL Inf product (pre/TIL) (Figure 5B and Supplemental Figure 8). Note, however, that the presence of preexisting NARTs that were further expanded did not appear sufficient to generate a clinical response, as we also observed pre/TIL NARTs in 3 patients with SD (Supplemental Figure 8). The perceived therapeutic benefit of preexisting NARTs that were further expanded may therefore relate more to the high frequency and persistence after expansion in selected patients than to their presence alone.

We observed that 62.5% (60 of 96) of NARTs observed in TIL Inf products were also detectable after ACT (Figure 5C). Furthermore, 57% of NARTs detected after ACT were novel and did not originate from the TIL Inf product (80 of 140), whereas 43% originated from the TIL Inf product (60 of 140; Figure 5C). These novel NARTs were transiently appearing and could represent epitope spreading. However, their appearances may not necessarily have therapeutic benefit, as they were observed across all RECIST groups (Figure 5, A and B) and present at lower frequency than newly engrafted NARTs (TIL NARTs present in post-ACT PBMCs) (Figure 5D). Finally, we observed that engrafted NARTs derived from the TIL Inf product (TIL plus pre/TIL) had a higher estimated frequency compared with their nonengrafted counterparts in the TIL Inf product (Figure 5E), suggesting engraftment to be associated with prior frequency.

To evaluate the impact of engrafted NART populations separately from that of nonengrafted and novel NARTs, appearing only in TIL Inf products and post-ACT PBMCs, respectively, we compared the diversity and frequency of engrafted NARTs (pre/TIL and TIL) in responders and nonresponders with available PBMCs throughout all time points (Figure 5, F and G). Interestingly, we observed that nonresponders had a markedly lower diversity (Figure 5F) and frequency (Figure 5G) of engrafted NARTs compared with responders in the first 2 sampling time points after ACT (<1 month and <4 months). These data suggest that responders were treated with TIL Inf products characterized by high-frequency, engrafting NARTs, whereas nonresponders were treated with TIL Inf products containing a relatively lower frequency of NARTs that were unable to engraft and persist after ACT. This is in line with prior TCR-sequencing efforts (29).

### The characteristics of immunogenic neoepitopes.

**Figure 6:**
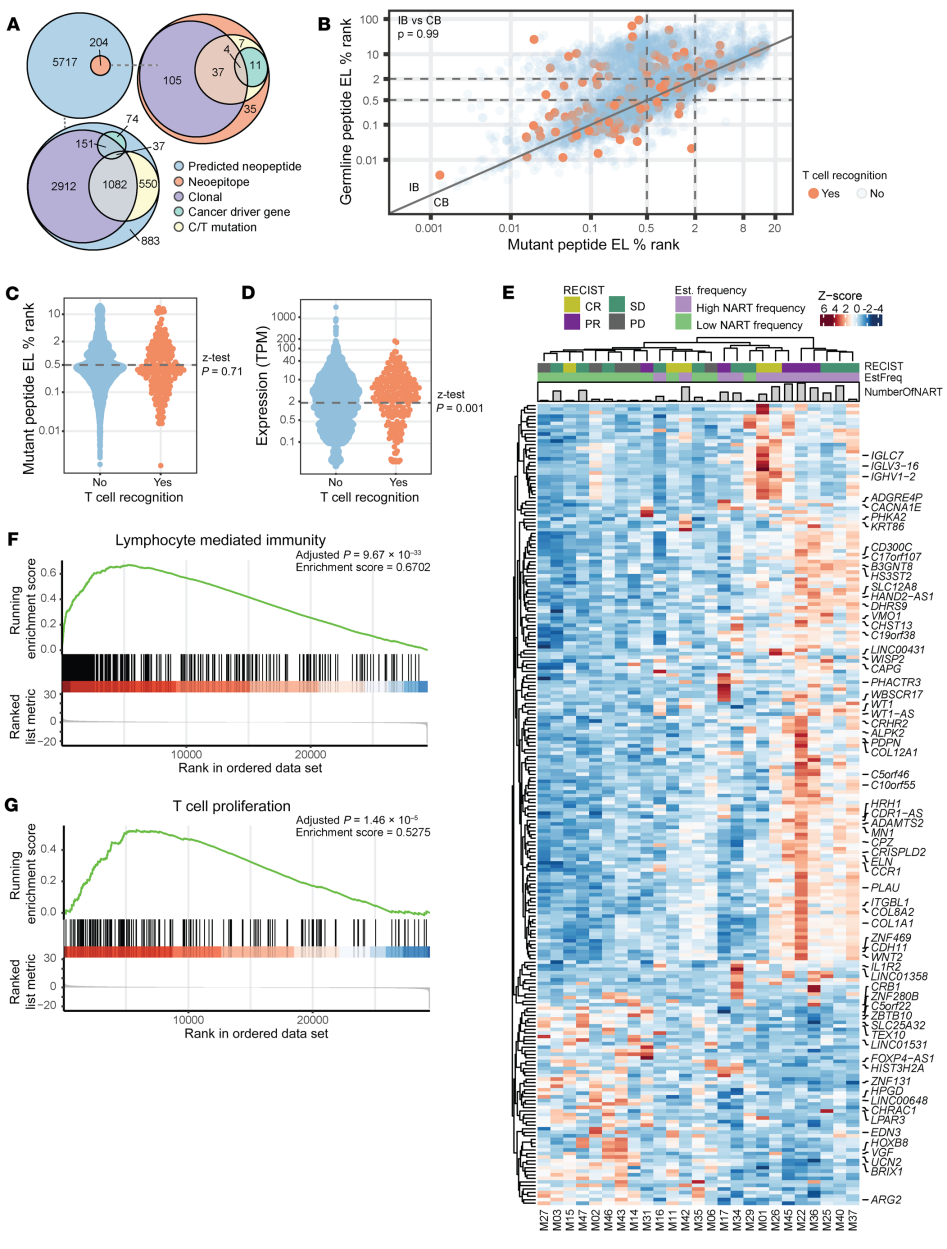
Characteristics of immunogenic neoepitopes. (**A**) Venn diagram of 5921 unique pMHC; 204 immunogenic and 5717 nonimmunogenic as determined by the presence of neoepitope-specific CD8^+^ T cells in patients at any time. The distribution and overlap of immunogenic versus nonimmunogenic neoepitopes deriving from either cancer-driver genes (6.5% versus. 3.3%, *P* = 0.0048, *z* test), C/T mutations (3.4% versus. 3.5%, *P* = 0.78, *z* test), or clonal mutations (80.1% versus 86.0% *P* = 0.03, *z* test). Clonality could not be determined for 913 neopeptides, as WES was performed on autologous tumor cell lines (M22, M24, and a subset of M15). These were excluded from the *z* test, but included in the Venn diagram as subclonal mutations for visualization. (**B**) Eluted ligand (EL) percentage rank score of mutated peptide compared with percentage rank score of the corresponding germline peptide without mutation or nearest germline peptide. Red, immunogenic peptides. 3.4% CB versus 3.5 % IB, *P* = 0.99, *z* test. (**C**) Mutant EL percentage rank score comparing proportion of immunogenic neoepitopes above and below 0.5 percentage rank score (3.3 % versus 3.5, *P* = 0.71, *z* test). (**D**) RNA expression (TPM) comparing proportion of immunogenic peptides with expression above and below 2 TPM (4.2 % versus. 2.6%, *P* = 0.001, *z* test). (**E**) Unsupervised clustering of the 226 differentially expressed gene according to high and low sum of estimated frequency within TIL Inf products split by the median frequency (0.63%). Denoted names were prioritized according to GO terms and known function. (**F**) Enriched GO gene set representing lymphocyte-mediated immunity. (**G**) Enriched GO gene set representing T cell proliferation. Significance threshold or GSEA was set at FDR ≤ 0.01. M24 was excluded from **D**–**G**, as RNA-Seq data were obtained from an autologous tumor cell line. *n* = 25. M22 was included in **D**–**G** using data from the tumor biopsy used for manufacturing of the infusion product.

Based on the large screen presented here, we evaluated T cell recognition against 5921 predicted neopeptides that were selected based on their HLA-binding characteristics and gene transcriptional levels in tumor next-generation sequencing (NGS) data. Of these predicted neopeptides, we detected specific CD8^+^ T cell recognition toward 204 neoepitopes in either TIL Inf products or PBMC samples from melanoma patients, while the remaining 5717 were not recognized by T cells in the evaluated patients (Figure 6A). The pool of immunogenic neoepitopes displayed characteristics related to both clonality and C/T mutations similar to that of the total library of evaluated neopeptides (Figure 6A). Hence, we did not observe a specific enrichment of T cell recognition toward clonal mutations, as has previously been suggested for non–small cell lung cancer (NSCLC) (32). Interestingly, cancer-driver genes (33) are significantly overrepresented in the fraction of immunogenic neoepitopes compared with the fraction of nonimmunogenic neopeptides (Figure 6A; 6.5% versus 3.3%, *P* = 0.0043). However, we did not find any immunogenic neoepitopes to be shared among patients, as has previously been observed in TILs isolated from colorectal cancer (34).

Our neopeptide library was preselected for predicted HLA binding. Within this pool, neoepitopes can be classified as either conserved binders (CBs), i.e., neopeptides with HLA binding similar to that of the mutated peptide versus the germ-line sequence, or improved binders (IBs), where the mutation affects HLA-binding capabilities, resulting in a neopeptide with improved HLA affinity compared with the germline sequence, as defined in Bjerregaard et al. (ref. 35 and Figure 6B). Immunogenic neoepitopes were represented in both categories, and we did not observe a significantly different distribution of immunogenic versus nonimmunogenic neopeptides among CBs versus IBs (3.4% CB versus. 3.5% IB, *P* = 0.99; Figure 6B). Furthermore, within the selected HLA affinity range evaluated here, we did not observe any further impact of HLA percentage rank score on neopeptide immunogenicity, evaluated as the potential enrichment of immunogenic neoepitopes below a percentage rank score of 0.5 (*P* = 0.71, *z* test; Figure 6C). In line with previous findings (34), we observed an enrichment of genes with RNA expression greater than 2 TPM among immunogenic neopeptides (Figure 6D; *P* = 0.001, *z* test).

TMB and predicted neoepitope load have previously been demonstrated as predictive for TIL-ACT outcome (5). We found a strong correlation between TMB and number of predicted neoepitopes (Supplemental Figure 9A). However, we did not find a correlation between TMB or the number of predicted neoepitopes and NART diversity and NART frequency (Supplemental Figure 9, B–E). This indicates that the presence of NARTs in TIL Inf products is an independent marker of therapeutic outcome in patients with metastatic melanoma. Since the interpatient variation in neopeptide library size may affect the correlation, we also correlated the number of NARTs detected and estimated frequency within the top 151 predicted neoepitopes so that it showed an equal representation of all patients (Supplemental Figure 9G). Again, no strong correlation was evident (Supplemental Figure 9, H–K), as multiple patients with low TMB showed medium-sized populations of neoepitope-specific CD8^+^ T cells in their respective TIL Inf products (Supplemental Figure 9G). This emphasizes the need to improve our predictive capacity for identification of those neoepitopes that give rise to functional T cell recognition and tumor cell killing and furthermore highlights that other parameters, beyond TMB, affect immune recognition.

The tumor microenvironment has a substantial influence on the capacity of the immune system to mount a T cell response toward the tumor and for such T cells to exert their function. Although the generation of TIL Inf products is conducted in vitro, the tumor microenvironment may still affect the capacity for T cell expansion and function. We used the available transcriptomic data from our neoepitope prediction pipeline as input for a differential gene expression analysis, grouping patients according to higher or lower than median sum of estimated NART frequency within TIL Inf products. From this, we observed 226 differentially expressed genes (Figure 6E), that were associated with 383 enriched Gene Ontology (GO) gene sets (36). The top 20 enriched GO gene sets were a collection of humoral and B cell–mediated mechanisms and several pathways pertaining to the immune cell signal transduction (Supplemental Figure 10). These gene sets are highly relevant in light of the recently revealed relationship among intratumoral lymphoid structures, antigen presentation, and therapeutic benefit following immunotherapy (37). Of further interest, we observed enriched presence of GO terms relating to lymphocyte-mediated immunity (Figure 6F) and increased T cell proliferation (Figure 6G).

## Discussion

Immune recognition and tumor killing by cytotoxic T cells are associated with a positive outcome across multiple immunotherapies (9, 32, 38); however, the presence of neoepitope-specific CD8^+^ T cells in TIL-ACT remains incompletely documented outside case responders (10–15). In the present study, we investigated the capacity of TIL Inf products to recognize predicted, HLA-binding neoepitopes originating from expressed, nonsynonymous mutations from 26 patients with metastatic melanoma. To this end, we utilized DNA barcode–labeled pMHC multimers from which we quantified NART diversity and frequency in TIL Inf products and patient PBMCs. We report recognition of a total of 106 neoepitopes within TIL Inf products from this cohort across all 4 RECIST groups. Supporting that the presence of NARTs affects the clinical response to TIL-ACT, we found that NART diversity and frequency were substantially lower in patients with PD when compared with patients with SD and PR and that NART frequency correlated with PFS and was higher in patients with clinical response to TIL-ACT (CR+PR).

We found that both NART diversity and frequency were highly variable across RECIST groups, especially within responding patients: 3 out of 11 CR/PR patients had 0 detectable NART populations. This variability could be due to limitations in neoepitope selection, contribution from other antigen types, insufficient HLA coverage, sampling bias, NART response frequencies below the threshold for detection (i.e., resulting in false-negative detection), or other NART-independent and/or HLA-I–independent pathways such as the MR1-dependent immune-recognition pathway (39).

Following each NART population from first appearance to last available PBMC time point further uncovered that responders were characterized by circulating NARTs of higher diversity and frequency in pretreatment PBMCs. This is interesting because pretreatment circulating NARTs could represent a biomarker for ongoing tumor recognition by CD8^+^ T cells, which, in extension, could provide a noninvasive way to measure immune activity of the tumor. However, identification of NARTs is a laborious and patient-specific process, and for biomarker purposes, a simpler measurement of NART reactivity should be developed. Responders were furthermore predominantly treated with TIL Inf products of high NART frequency capable of engrafting and persisting after TIL-ACT at an estimated frequency higher than 0.01%. Additionally, we observed that engrafted NARTs initially appeared with an overall higher estimated frequency in the TIL Inf product compared with nonengrafted NARTs, which indicates that successful NART expansion precedes successful engraftment. As mentioned, the persistence of tumor antigen–specific TCRs has been hypothesized to drive therapeutic benefit following TIL-ACT (29). Interestingly, this hypothesis has recently been supported in the metastatic melanoma setting (40), where the persistence of neoantigen-specific TCRs after TIL-ACT correlated with CD39^–^CD69^–^ stem-like T cells capable of self-renewal, differentiation, and further expansion upon stimulation. Future efforts to discover and quantify the presence of NARTs may benefit from a simultaneous characterization of stem-like phenotypes to increase our understanding of why certain NARTs are superior in their capacity for expansion and persistence. Together with our current report, this identifies an unmet need to improve the manufacturing of TIL Inf products to increase the frequency of tumor-specific CD8^+^ T cells that are able to engraft and persist in patients after ACT.

Interestingly, we observed that 2 out of 3 patients with PD and multiple patients with SD appeared to have NARTs in peripheral blood despite the lack of persisting NART populations in the TIL Inf product. This suggests that selected nonresponders had ongoing tumor recognition that was not expanded by the TIL-manufacturing process (i.e., failure to expand meaningful NARTs), perhaps due to poor tumor immune infiltration (i.e., immunologically “cold”). Thus, development of technologies to expand tumor-specific CD8^+^ T cells from peripheral blood may be beneficial for the future treatment of patients that do not benefit from conventional TIL-ACT. Given information on the antigen recognized in peripheral blood, other strategies, such as therapeutic vaccination (41, 42), could furthermore be combined to increase the likelihood of generating long-lasting CD8^+^ and CD4^+^ memory T cells from TIL-ACT.

We additionally observed novel NARTs at multiple time points after infusion in both responders and nonresponders. This might illustrate epitope spreading as a result of tumor-cell killing in responders. However, these late-emerging NART populations were present at a lower frequency and appeared to be more transient than those transferred in the TIL Inf product. Thus, epitope spreading, with T cell recognition of preexisting mutations and their derived peptide products, does not appear to play a major role following TIL-ACT. However, this does not exclude a potential therapeutic role for epitope spreading based on T cell recognition toward novel mutations occurring after immunotherapy.

Finally, we observed that lymphocyte activity and proliferation within the tumor microenvironment were associated with higher NART frequency in TIL Inf products, suggesting that ongoing immune activity within the tumor supports the manufacturing of TIL Inf products containing a high frequency of NARTs. Superior T cell proliferation and response to checkpoint inhibition is associated with intratumoral tertiary lymphoid structures, which maintain a niche of professional antigen-presenting cells and proliferating T cells (37, 43). Tertiary lymphoid structures could, therefore, possibly support the successful expansion of TILs prior to successful TIL-ACT. However, the relationship among ongoing T cell proliferation, successful TIL expansion, and therapeutic response remains undetermined.

Both TIL expansion and posttransfer persistence of CD8^+^ NARTs may additionally be affected by supporting CD4^+^ T cells (44). So far, no differences have been observed between CD8-enriched TIL products and TIL products containing different lymphocytes (although the majority are CD8; ref. 45). Furthermore, epitope spreading as evaluated here for CD8^+^ T cells may likewise occur for CD4^+^ T cells, and further insight into the relationship between CD4^+^ and CD8^+^ tumor-reactive T cells and the relevance for shared antigen recognition are critical aspects for addressing future improvements in immunotherapy. However, technical limitations still prohibit detailed epitope mapping of CD4^+^ NARTs, as conducted here for CD8^+^ NARTs (46).

In this study, we screened for recognition among 5921 predicted neopeptides arising from nonsynonymous mutations, of which we found recognition of 1.8% (106 neoepitopes) in TIL Inf products and additionally 98 neoepitopes in peripheral blood before or after TIL Inf, making a T cell recognition percentage of 3.4%. This illustrates that neoepitope prediction is feasible, but it remains a cumbersome approach to identifying neoepitope-specific CD8^+^ T cells in metastatic melanoma. While recent efforts have led to significant improvements in the prediction of antigen processing and HLA binding (47), a gap remains in our ability to predict which of the presented neoepitopes are able to give rise to T cell recognition (21). Among the neoepitopes recognized by T cells in this study, we observed an enrichment of neoepitopes derived from cancer-driver genes and genes expressed above 2 TPM. However, despite these characteristics, the majority of the neoepitopes detected were derived from passenger mutations, and no stringent criteria could be assigned to determine the neoepitopes driving T cell recognition.

In conclusion, our study describes the critical contribution of NARTs to the clinical outcome in TIL-ACT therapy and provides a thorough characterization of neoantigens recognized by T cells in this therapeutic context. To this end, our study highlights a critical need for improving TIL-ACT manufacturing and the capacity to predict immunogenic neoepitopes. Strategies to improve the expansion and engraftment of NARTs in TIL Inf products should further improve clinical outcome.

## Methods

### Patient material.

To study the role of NARTs in TIL-ACT in melanoma, we evaluated 26 patients with unresectable or metastatic melanoma enrolled in a phase I/II clinical study of ACT (ClinicalTrials.gov NCT00937625). Demographic and clinical information for each patient ID are available in previous reports (4, 5, 26). TIL Inf products were generated by expanding TILs in vitro from tumor lesions following a rapid expansion protocol (REP) with high-dose IL-2, as described previously (48). All patients were included at the time of progression from previous treatment or treatments with either IL-2/IFN-α and/or anti–CTLA-4 treatment and/or DC vaccination and/or temozolomide and/or vemurafenib (26). Furthermore, as specified previously, 4 patients received vemurafenib between surgical resection and TIL-ACT (M27, M29, M35, M36; ref. 4). Clinical response was assessed according to RECIST 1.0. Among the 26 patients, 5 were CRs, 6 were PRs, 10 were SD, and 5 were PD patients (4), with a median PFS and OS of 3.85 and 23.25 months, respectively. Using DNA barcode–labeled pMHC multimers, we analyzed the TIL Inf products from all 26 patients for neoepitope-specific CD8^+^ T cells. From 19 of these patients, we additionally analyzed corresponding PBMC samples before and at multiple time points after TIL-ACT (Supplemental Table 1). Tumor sequencing data (RNA and WES) were available from 26 of the 27 patients enrolled in the trial. PBMCs from healthy donors were obtained from whole blood by density centrifugation on Lymphoprep in Leucosep tubes and cryopreserved at −150°C in FCS (Gibco, Thermo Fisher Scientific) plus 10% DMSO.

### TIL sorting and expansion.

Young TILs were thawed and cultured overnight at 37°C in complete medium (CM) (RPMI-1640 supplemented with 10% heat-inactivated human serum), 100 U/mL penicillin, 100 μg/mL streptomycin, 1.25 μg/mL fungizone, and 6000 IU/mL IL-2. Cells were washed twice in R0 (RPMI 1640, 100 U/mL penicillin, 100 μg/mL streptomycin) and stained with 0.2 μg of pMHC tetramers for 10 minutes at 37°C. Tetramers were assembled from fluorescent-streptavidin conjugates (PE, catalog 405204, BioLegend; APC, catalog 405243, BioLegend; BV421, catalog 563259, BD) and biotinylated, recombinant UV-cleavable pMHC-1 (23, 24). An empty disulfide-stabilized monomer was used for A*02:01-Y84C (49). Anti–CD4-FITC (clone SK3, catalog 345768, BD) and anti–CD8-PerCP (clone SK1, catalog 345774, BD) antibodies were added for a further 20 minutes at 37°C. Cells were washed with R0, resuspended in R0 plus 10% heat-inactivated human serum, and sorted by flow cytometry using the BD FACSAria cell sorter (BD Biosciences) into a 96-well plate. Sorted CD8^+^ tetramer^+^ cells were expanded in 2 consecutive minirapid expansions 9 days apart, on day 0 and day 9. The second minirapid expansion was omitted in cases with abundant proliferation. In brief, 5 × 10^5^ allogeneic feeder cells from healthy donors, 30 ng/mL anti-CD3 antibody (clone OKT3, Janssen-Cilag), master mix made of 50% CM and 50% rapid expansion medium (RM) consisting of AIM-V medium (Gibco, Thermo Fisher Scientific), and 1.25 μg/mL fungizone supplemented with 6000 IU/mL IL-2 with 10% heat-inactivated human serum (HS) were added to sorted cells and cultured at 37°C; 50% of the media (without OKT-3) was replaced after 5 days and subsequently every 2 days.

### Intracellular cytokine assay.

Tumor cells were either pretreated with IFN-γ (100 IU/mL, Peprotech) or left untreated for 3 days. TILs were then added in a 1:1 ratio, with protein transport inhibitors brefeldin A (1:1000 dilution, GolgiPlug, catalog 555029, BD), Monensin (1:1000 dilution, GolgiStop, catalog 554724, BD), and anti–CD107a-BV421 antibodies (clone H4A3, BD 562623). Tumor cells and TILs were cocultured for 5 hours, after which all cells were stained with Near-IR LIVE/DEAD (Life Technologies) and for surface markers CD3-FITC (clone SK7, BD 345764), CD8-QDot605 (clone 3B5, Thermo Fisher Q10009), and CD4-BV711 (clone SK3, BD, catalog 563028). Subsequently, the cells were fixed and permeabilized (eBioscience) overnight and stained for intracellular cytokines TNF-APC (clone MAb11, BD catalog, 554514) and IFN-γ–PE-Cy7 (clone B27, BD, catalog 557643). Cells were analyzed on a Novocyte Quanteon (ACEA Biosciences). See details related to antibodies used in Supplemental Table 2.

### Neoepitope prediction.

WES and RNA-Seq data were obtained from digested tumor fragments, except for M22 and M24, for which autologous tumor cell lines were used. Two WES files from M15 were utilized and their results combined, one from an autologous tumor digest and another from an autologous tumor cell line. All WES data were obtained from tumor material from the same biopsy as was used for manufacturing of the corresponding TIL Inf products, expect for M22, for which the tumor cell line was derived from an earlier time point. FASTQ files from WES and RNA-Seq were preprocessed using Trim Galore (50), version 0.4.0. WES reads were aligned to the human genome (GRCh38) using Burrows-Wheeler Aligner (51), version 0.7.15, with default mem parameters, and duplicate reads were marked using MarkDuplicates from Picard Tools (52), version 2.9.1. Peptides were extracted and prioritized using MuPeXI (20), version 1.1.3, and netMHCpan, version 4.0, (22), providing as input the somatic variants obtained following GATK, version 3.8.0, best practices, the RNA-Seq expression values calculated using Kallisto, version 0.42.1 (53), and the HLA alleles inferred from normal WES samples using OptiType, version 1.2 (54). For patients with high neoantigen load, all predicted neoepitopes with a percentage rank score of 0.5 or less and TPM of 0.1 or more were included. For patients with lower neoantigen load, we lowered the expression threshold to 0.01 TPM or more and selected the top 200 predicted neopeptides according to percentage rank score. All predicted neopeptides and virus control peptides were synthesized and purchased from Pepscan (Pepscan Presto) and dissolved to 10 mM in DMSO.

For each cancer-specific nonsynonymous mutation, the HLA-I–binding potential of mutation-derived peptides was predicted using netMHCpan, version 4.0 (20, 22). For each patient, a minimum of 200 top-ranking neopeptides were included. The ranking was based on the predicted HLA-I binding (percentage rank score) and the transcription of the corresponding gene as RNA TPM.

### Clonality.

Copy number, purity, and ploidity were found using Sequenza, version 3.0 (55). As input, printed reads from normal and tumor were used as input to Sequenza. Sequenza-utils, version 3.0, bam2seqz with GRCh38 was used as a reference. To run the Sequenza copy number call with GRCh38, the R packages Shixiang/copynumber, version 1.26.0 (56), was applied. The created seqz files were used as input to sequenza-utils seqz_binding, and the outputs were used to Sequenza utils snp2seqz. To reduce the amount of false negatives according to the built-in mutations called from Sequenza, copy number files from the mutect2 output were merged with the copy number call from the bam files. Sequenza results and PyClone inputs were generated with the Sequenza packages in R, version 3.6.1. To find clonal mutations, PyClone, version 0.13.0 (57), was applied with the cellularity given from Sequenza and max cluster of 30 and minimum size of 0 to get all possible mutations given. Clonal mutations were filtered with a cluster size of minimum 80 and cellularity of minimum 90. Clonality could not be computed for M22, M24, and part of M15, as the underlying WES data came from autologous tumor cell lines.

### Generation of DNA barcode–labeled pMHC multimers.

Oligonucleotides containing distinct 25 mer nucleotide sequences (58) were purchased from LGC Biosearch Technologies. All oligos carry a 6 nt unique molecular identifier (59). Oligonucleotides modified with a 5′ biotin tag (oligo A) were joined to unmodified, partially complementary oligonucleotides (oligo B) to generate more than 1000 unique double-stranded AxBy DNA barcodes. Combinations of A and B oligos (one of each) were mixed with 5× Sequenase Reaction Buffer Mix (PN 70702, Affymetrix) to final concentrations of 26 μM (oligo A) and 52 μM (oligo B), respectively, heated to 65°C for 2 minutes, and allowed to anneal by cooling slowly to less than 35°C over 15 to 30 minutes. The annealed oligo As and Bs were elongated to create double-stranded AxBy DNA barcodes by adding Sequenase polymerase (70775Y, Affymetrix), 20 μM DTT, and 800 μM or 72 μM dNTPs, followed by incubation for 5 to 10 minutes at room temperature. Elongated AxBy barcodes were diluted in nuclease-free water plus 0.1% Tween to 2.17 μM (with respect to the A oligo) and stored at −20°C. Attachment of 5′ biotinylated AxBy DNA barcodes to PE- and streptavidin-conjugated dextran (Fina Biosolutions) was performed by mixing the 2 components at final concentrations of 154 nM dextran backbone and 77 nM barcode in order to obtain 0.5 barcodes for each dextran backbone and subsequent incubation for 30 minutes at 4°C.

Refolded, biotinylated pMHC-I was subsequently added at a stoichiometry of approximately 16.5 pMHC molecules per dextran; these were generated through UV-mediated exchange of cleavable ligands as described previously (23, 24). In brief, MHC monomers bound to UV-sensitive ligands were mixed with HLA-matching peptides at a final concentration of 50 μg/mL monomer and 100 mM peptide and exposed to UV light for 60 minutes (366 nm). Afterwards, pMHC monomers were centrifuged for 5 minutes at 3300*g* and then coupled to DNA barcode– and PE-labeled dextran backbones to a final concentration of 35 μg/mL monomer and 4.2 × 10^−8^ M barcode- and PE-labeled dextran backbone and incubated for 20 minutes on ice. Then a freezing buffer was added to reach PBS plus 0.5% BSA plus 100 μg/mL herring DNA plus 2 mM EDTA plus 5% glycerol and 909 nM d-biotin, and after 20 minutes on ice, the pMHC multimers were stored at −20°C until use.

### T cell staining with barcode-labeled pMHC multimers.

Cryopreserved cells were thawed, washed twice in RPMI plus 10% FCS, and then washed in barcode-cytometry buffer (PBS plus 0.5% BSA plus 100 μg/mL herring DNA plus 2 mM EDTA). Before staining, MHC multimers were thawed on ice, centrifuged for 5 minutes at 3300*g*, and 1.5 μL (0.043 μg) of each distinct pMHC was taken from each well, avoiding potential aggregates in the bottom, and pooled. The volume of the reagent pool was reduced by ultrafiltration to obtain a final volume of approximately 80 μL of pooled MHC multimers per staining. Centrifugal concentrators (Vivaspin 6, 100,000 Da, Sartorius) were saturated with BSA before use. Following ultrafiltration, the pool of multimers was spun at 10,000*g* for 2 minutes to sediment potential aggregates. An aliquot of approximately 5 μL of the MHC multimer reagent pool was stored at −20°C for later baseline analysis. Up to 10 million cells were stained in 80 μL with 50 nM dasatinib and multimer pools in a 15-minute incubation at 37°C. Following incubation, the cells were stained with an antibody mix containing CD8-BV480 (clone RPA-T8, BD, catalog 566121), dump channel antibodies (CD4-FITC (clone SK3, BD, catalog 345768), CD14-FITC (clone MϕP9, BD, catalog 345784), CD19-FITC (clone 4G7, BD, catalog 345776), CD40-FITC (clone LOB7/6, Serotech, catalog MCA1590F), and CD16-FITC (clone NLP15, BD, catalog 335035), and a dead cell marker (LIVE/DEAD Fixable Near-IR; Invitrogen L10119) and incubated for 30 minutes at 4°C. Samples were stained with antibodies in a total volume of 100 μL. See staining concentrations in Supplemental Table 2. Cells were washed 3 times in barcode cytometry buffer and fixed in 1% paraformaldehyde (PFA) for 0.5 to 24 hours before they were washed twice and resuspended in barcode-cytometry buffer. Cells were acquired within a week after multimer staining.

### Sorting of pMHC multimer^+^ T cells.

Multimer-binding CD8^+^ T cells were sorted on a FACSAria Fusion or FACSMelody Cell Sorter (BD) into BSA-saturated tubes containing 100 μl of barcode/cytometry buffer. We gated on single, live, CD8^+^, and dump channel–negative (CD4, CD14, CD16, CD19, and CD40) lymphocytes and sorted all multimer-positive PE cells within this population. As tested and described in Bentzen et al. (19), inclusion of CD8^+^ multimer negative cells in the sorting gate does not have an impact on the final results because the fluorescence signal is used only for sorting out the relevant cells. Determination of antigen specificity is done solely based on the DNA barcode. The sorted cells were centrifuged for 10 minutes at 5000*g*, and the buffer was removed. The cell pellet was stored at −80°C. The percentage of multimer^+^ CD8^+^ T cells was used as input for estimation of epitope-specific CD8^+^ T cells (see *Processing of sequencing data from DNA barcodes*). Three samples were run without exported flow cytometry files, precluding adequate estimation of frequency after sequencing of DNA barcodes (M15, TIL Inf product; M40, pre-ACT PBMCs; and M40, PBMCs <1 month). TIL Inf product from M47 was stained again to estimated percentage of multimer^+^ CD8^+^ T cells. M15 had no significant hits among barcoded multimers (i.e., sum of estimated frequency was set to 0%). See antibody assay details in Supplemental Table 2.

### DNA barcode amplification.

DNA barcode amplification was performed using Taq PCR Master Mix Kit (QIAGEN, 201443) and 3 μM of forward and reverse primers (LGC Biosearch Technologies). PCR amplification was conducted on sorted multimer-binding T cells (in <19 μL of buffer) and on a triplicate of the stored aliquot of the MHC multimer reagent pool (diluted 10.000× in the final PCR) under the following conditions: 95°C for 10 minutes; 36 cycles: 95°C for 30 seconds, 60°C for 45 seconds, 72°C 30 for seconds, and 72°C for 4 minutes. The multimer reagent pool was used as the baseline to determine the number of DNA barcode reads within a nonprocessed MHC multimer reagent library. PCR products were purified with a QIAquick PCR Purification Kit (QIAGEN)m and the amplified DNA barcodes were sequenced at PrimBio using an Ion Torrent PGM 316 or 318 chip (Life Technologies).

### Processing of sequencing data from DNA barcodes.

Sequencing data were processed by the software package Barracoda, available online (https://services.healthtech.dtu.dk/service.php?Barracoda-1.8). This tool identifies the barcodes used in a given experiment, assigns PCR used sample IDs and pMHC specificity to each barcode, and counts the total number (clonally reduced) of reads for each DNA barcode. Furthermore, it accounts for barcode enrichment based on methods designed for the analysis of RNA-Seq data, implemented in the R package edgeR; specifically, log_2_ fold changes (FCs) in read counts mapped to a given sample relative to the mean read counts mapped to triplicate baseline samples are estimated using normalization factors determined by the trimmed mean of M values method. Enriched barcodes were regarded as significant when the adjusted *P* value was below 0.001, which equals an FDR< 0.1 (estimated using the Benjamini–Hochberg method). Barracoda outputs were further processed and annotated using R 4.0.2 — adding relevant clinical information and excluding signals arising from insufficient read depth (percentage of read count < 0.1). Furthermore, biologically relevant barcode enrichment was defined as an estimated frequency of 0.01% or more and without presence in partially HLA-matching healthy donor PBMCs. 227 Multimers were excluded due to technical concerns regarding HLA-C*05:01 (M22, 140 multimers; M27, 46 multimers) and HLA-C*02:02 (M43, 41 multimers). Peptide missannotations, which originated from pipetting errors discovered through cross referencing of ordering and annotation tables (M27, 1 multimer; M35, 40 multimers; M46, 1 multimer), were also excluded. Frequency of a pMHC-specific CD8^+^ T cell population was estimated based on the percentage of read count of the associated barcode out of the total percentage of the multimer-positive CD8^+^ T cell population. Sum of estimated frequency represents the pooled frequencies of all T cell populations in a given sample. Due to differences in number of producible HLA molecules, the number and frequency of neoepitope-specific CD8^+^ T cells were normalized to the mean absolute HLA coverage in the cohort: (average HLA covered [across all panels]/HLA covered [patient panel]).

### Structural analysis of overlapping mutated peptides and HLA binding.

Structural pMHC models were generated using the method described in TCRpMHCmodels (60). All peptides were bound to HLA-A*01:01 and the sequence for this MHC molecule was downloaded from the IMGT database (61). To get the electrostatic potential for each of the pMHC models, hydrogen atoms were added using the phenix.reduce protocol previously described (62), after which Delphi (63) was used to calculate the electrostatic potential with the following parameters: scale = 1.0, perfil = 70.0, indi = 4.0, exdi = 80.0, prbrad = 1.4, salt = 0.15, ionrad = 2.0, bndcon = 2, linit = 800, maxc = 0.0001, sigma = 2.0, srfcut = 20.0 and gaussian = 1. The electrostatic potential from Delphi was finally virtualized using PyMOL (https://pymol.org/).

### Differential expression analysis.

RNA-Seq data for differential gene expression analysis exclusively came from tumor digests, i.e., no autologous tumor cell lines were used. Output files from Kallisto were used as input to DESeq2, version 1.26.0, from R/bioconductor with default options (64) to find differential expressed genes (adjusted *P* < 0.05, related to high and low sum of estimated frequency split by the median and PFS split by equal or below the median). GO enrichment analysis was performed using R, version 4.0.2, with the packages enrichplot, version 1.11.0.991 (65), and clusterProfiler, version 3.16.1, with Benjamini-Hochberg at *P* value adjustment (36).

### Code availability.

MuPeXi used for neoepitope prediction is available for all users at https://services.healthtech.dtu.dk/service.php?MuPeXI-1.1 and has been published (20). Visualization of pMHCs was generated as described in Methods. Analysis of DNA barcodes was performed as described in Methods, and the bioinformatics pipeline is available (https://services.healthtech.dtu.dk/service.php?Barracoda-1.8). Code used for further analysis and visualization was written in R as performed as described in methods.

### Statistics.

Statistical analysis of DNA barcoding data was performed using the software package Barracoda as described above. Survival analysis used percentiles and medians (number of NARTS or frequency) to define thresholds to split the cohort. Any values matching the threshold were treated as belonging to the lower group. Mantel-Cox test was used to evaluate the effect of NARTs on survival, and HRs were calculated using the log-rank approach with GraphPad Prism 8. Correlations were tested using nonparametric, 2-sided Spearman’s correlation test, except for Supplemental Figure 1D, where we used a 2-sided Pearson’s correlation. Two-sided *z* tests (prop.test) were applied where specified for Figure 6, A, C, and D. All 2-group comparisons were performed using nonparametric Mann-Whitney *U* test with a significance threshold of 0.05. Multigroup comparisons were performed by an initial nonparametric Kruskal-Wallis test followed by post hoc Dunn’s multiple comparison test.

### Study approval.

This study was conducted using TILs and PBMCs from patients enrolled in a clinical study (ClinicalTrials.gov NCT00937625). All patients signed a written consent form according to the Declaration of Helsinki. The study was approved by the local ethics committee for the capital region of Denmark (Region H). Likewise, healthy donor samples were collected by approval of the local Scientific Ethics Committee for the capital region of Denmark (Region H), with donor written, informed consent obtained according to the Declaration of Helsinki. Healthy donor blood samples were obtained from the blood bank at Rigshospitalet, Copenhagen, Denmark. All samples were obtained anonymously

## Author contributions

NPK, CH, and SAT performed experiments, analyzed data, generated figures, and wrote the manuscript. AB conducted all bioinformatics analyses and generated figures. AD and MDC performed experiments and analyzed data. IC predicted neoepitopes. KKM conducted structural analyses of pMHC and generated figures. JSH assisted neopeptide selection and pMHC multimer production. AMB assisted neopeptide prediction. AKB provided technical guidance. AMM supported data analyses. ZS codesigned in silico platforms and supported funding. NM assisted in bioinformatics guidance. RA provided patient material, diagnosed and characterized patients, and generated tumor cell lines. MN designed in silico platforms and supervised neoepitope prediction. GBJ conducted sequencing analysis and discussed data. MD provided patient material, cosupervised the study, and discussed data. IMS provided patient material, cosupervised the study, discussed data, and revised the manuscript. SRH conceived the concept, supervised the study, discussed data, and wrote the manuscript. NPK, CH, and SAT are listed as co–first authors. NPK led the effort through the revision phase and is therefore listed first. CH and SAT are listed in alphabetical order.

## Supplementary Material

Supplemental data

ICMJE disclosure forms

## Version 1 11/23/2021

In-Press Preview

## Version 2 01/18/2022

Electronic publication
