# Supplementary material for: Neoantigen-reactive CD8+ T cells affect clinical outcome of adoptive cell therapy with tumor-infiltrating lymphocytes in melanoma
Source: J Clin Invest. 2022 Jan 18;132(2):e150535. doi: 10.1172/JCI150535 (PMC8759789; doi:10.1172/JCI150535)
Supplement: Supplemental data [file jci-132-150535-s180.pdf]

Supplementary Materials

- **Supplemental Table 1:** Availability of peripheral blood samples pre- and post-ACT in melanoma patient cohort.
- **Supplemental Table 2:** Data reporting for fluorochrome-conjugated antibodies used in flow cytometry.
- **Supplemental Figure 1:** Supporting information regarding neopeptide multimer libraries.
- **Supplemental Figure 2:** Detection of NARTs in TIL Inf products from two melanoma patients.
- **Supplemental Figure 3:** Detection of NARTs specific for $AKAP^{P1796L}$ peptide variants ('SILSY' variants).
- **Supplemental Figure 4:** Tumor recognition by expanded, $AKAP^{P1796L}$-specifc NARTs.
- **Supplemental Figure 5:** Tumor-mutational burden, estimated frequency and diversity of neoepitope-specific CD8 T cells in TIL-ACT.
- **Supplemental Figure 6:** Impact of NART diversity and frequency, as well as presence of immunogenic mutations on overall and progression-free survival.
- **Supplemental Figure 7:** NARTs in TIL samples and peripheral blood over time.
- **Supplemental Figure 8:** Distribution and temporal appearance of NARTs in TIL-ACT treated patients.
- **Supplemental Figure 9:** NART diversity and frequency within TIL Inf products does not correlate with tumor mutational burden (TMB) or number of predicted neoepitopes.
- **Supplemental Figure 10:** Exploratory analysis of differentially expressed genes.

Patient ID	PBMC pre-ACT<br>8 days prior	TIL Inf Prod	PBMC post-ACT<br>< 1 month	PBMC post-ACT<br>< 4 months	PBMC post-ACT<br>< 12 months	PBMC post-ACT<br>< 24 months	PBMC post-ACT<br>< 48 months
**M01**		√			√		√
**M14**	√	√	√	√			
**M17**	√	√	√		√	√	√
**M22**	√	√	√	√	√	√	√
**M24**	√	√	√	√	√	√	√
**M25**	√	√	√	√			
**M26**	√	√	√	√	√	√	√
**M27**	√	√	√	√			
**M29**	√	√	√	√			
**M31**	√	√	√	√	√		
**M34**	√	√	√				
**M35**	√	√	√				
**M36**	√	√	√	√	√		
**M40**	√	√	√		√		
**M42**	√	√	√	√	√	√	√
**M43**	√	√	√	√	√		
**M45**	√	√	√	√	√		
**M46**	√	√	√	√	√		
**M47**	√	√	√	√			

Supplemental Table 1. Availability of peripheral blood samples pre- and post-ACT in melanoma patient cohort.

Marker	Assay	Fluorochrome	Clone	Supplier	Cat. Number	Staining conc.
CD8	Multimer screening	BV480	RPA-T8	BD	566121	2:100
CD4	Multimer screening	FITC	SK3	BD	345768	1.25:100
CD14	Multimer screening	FITC	MφP9,	BD	345784	3.13:100
CD16	Multimer screening	FITC	NLP15	BD	335035	1.56:100
CD19	Multimer screening	FITC	4G7	BD	345776	6.25:100
CD40	Multimer screening	FITC	LOB7/6	Serotech	MCA1590F	2.5:100
CD4	TIL sorting	FITC	SK3	BD	345768	240ng/ul
CD8	TIL sorting	PerCP	SK1	BD	345774	500ng/ul
CD107a	ICS	BV421	H4A3	BD	562623	0.3:50
CD3	ICS	FITC	SK7	BD	345764	5ng/ul
CD8	ICS	QDOT605	3B5	Thermo Fischer	Q10009	0.2:50
CD4	ICS	BV711	SK3	BD	563028	1:50
TNFα	ICS	APC	Mab11	BD	554514	4ng/ul
IFNγ	ICS	PE-Cy7	B27	BD	557643	1.5:50

Supplemental Table 2: Data reporting for fluorochrome-conjugated antibodies used in flow cytometry. ICS, Intracellular Cytokine Staining.

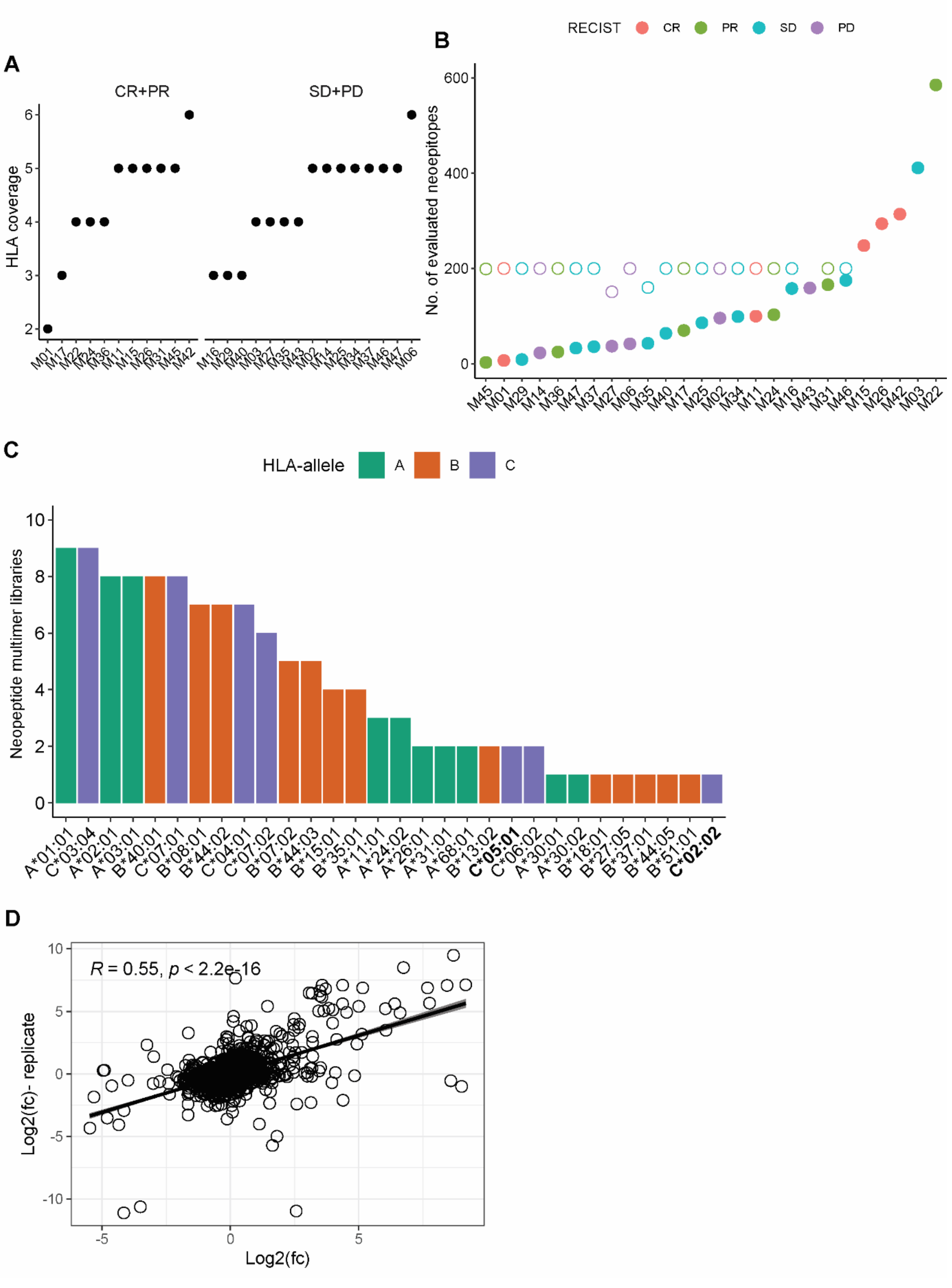
A
CR+PR
SD+PD
HLA coverage
M01 M17 M22 M24 M36 M11 M15 M26 M31 M45 M42 M16 M29 M40 M03 M27 M35 M43 M02 M14 M25 M34 M37 M46 M47 M06
B
RECIST CR PR SD PD
No. of evaluated neoepitopes
M45 M01 M29 M14 M36 M47 M37 M27 M06 M35 M40 M17 M25 M02 M34 M11 M24 M16 M43 M31 M46 M15 M26 M42 M03 M22
C
HLA-allele A B C
Neopeptide multimer libraries
A*01:01 C*03:04 A*02:01 A*03:01 B*40:01 C*07:01 B*08:01 B*44:02 C*04:01 C*07:02 B*07:02 B*44:03 B*15:01 B*35:01 A*11:01 A*24:02 A*26:01 A*31:01 A*68:01 B*13:02 C*05:01 C*06:02 A*30:01 A*30:02 B*18:01 B*27:05 B*37:01 B*44:05 B*51:01 C*02:02
D
R = 0.55, p < 2.2e-16
Log2(fc)- replicate
Log2(fc)

Supplemental Figure 1. Supporting information to neopeptide multimer libraries. (**A**) HLA coverage per patient. Number of HLAs evaluated for each patient-specific multimer library. (**B**) Neopeptide library size. Closed circles represent the number of predicted neoepitopes that can bind producible HLAs with %rank $\leq$ 0.5, and expression (TPM) $\geq$ 0.1. Open circles represent the number of evaluated multimers (see methods). Note, that hollow circles are overlaid by filled circles for M43, M15, M26, M42, M03, and M22. (**C**) HLA alleles and their prevalence in assembled multimer libraries. Bold: excluded HLA-alleles due to technical issues. (**D**) Correlation of replicate multimer screens in TIL Inf samples of nine patients. Shown is the log2(fc) change of barcode read counts compared to triplicate panel baseline. Normality was tested using Shapiro-Wilk's method followed by Pearson correlation. Grey shading represent the 95% confidence intervals.

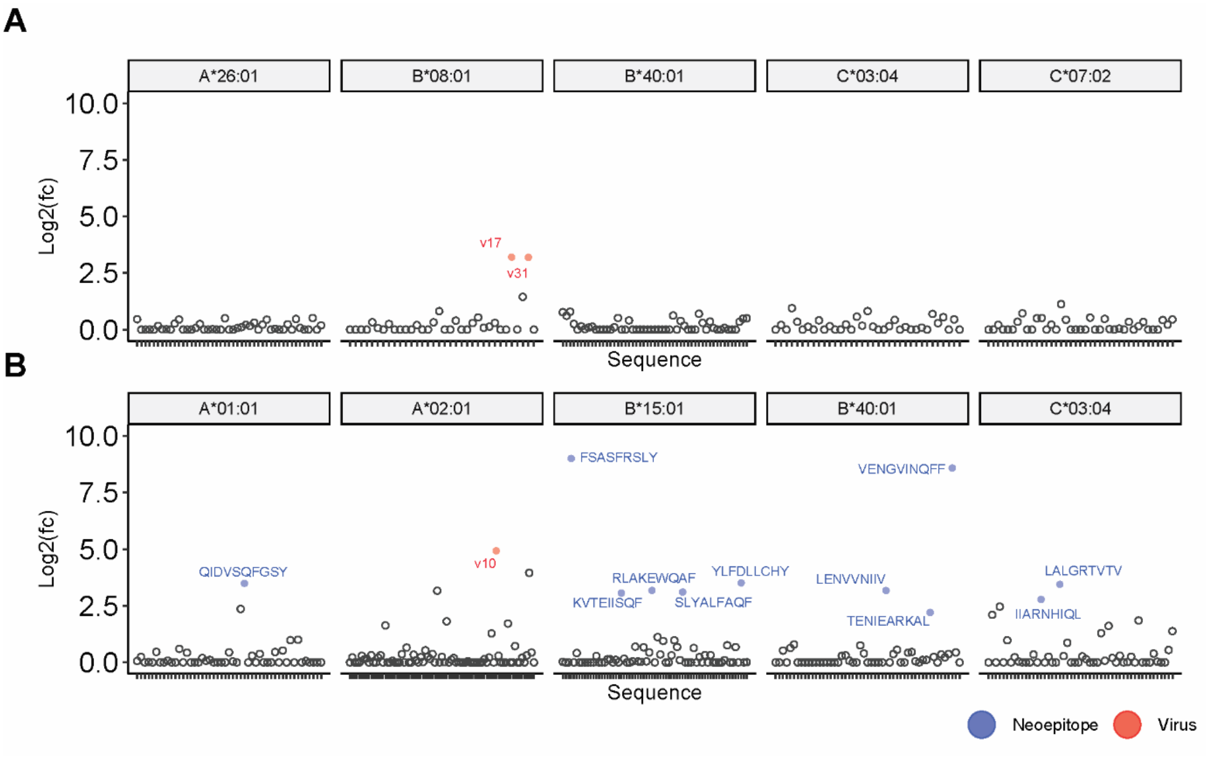

Supplemental Figure 2. Detection of NARTs in TIL Inf products from two melanoma patients. Example of a full screening using barcoded pMHC multimers for detection of both neoepitope-and virus-epitopes specific CD8+ T cells in TIL Inf products for melanoma patient M14 (PD) (**A**) and M26 (CR) (**B**). The data is separated according to the HLA alleles included in the screen. Blue: Virus-specific CD8 T cells. Red: NARTs. Black: Non-enriched barcodes. V10 annotate FLU peptide FLYALALLL, v17 annotate EBV peptide RAKFKQLL, and v31 annotate EBV virus peptide FLRGRAYGL.

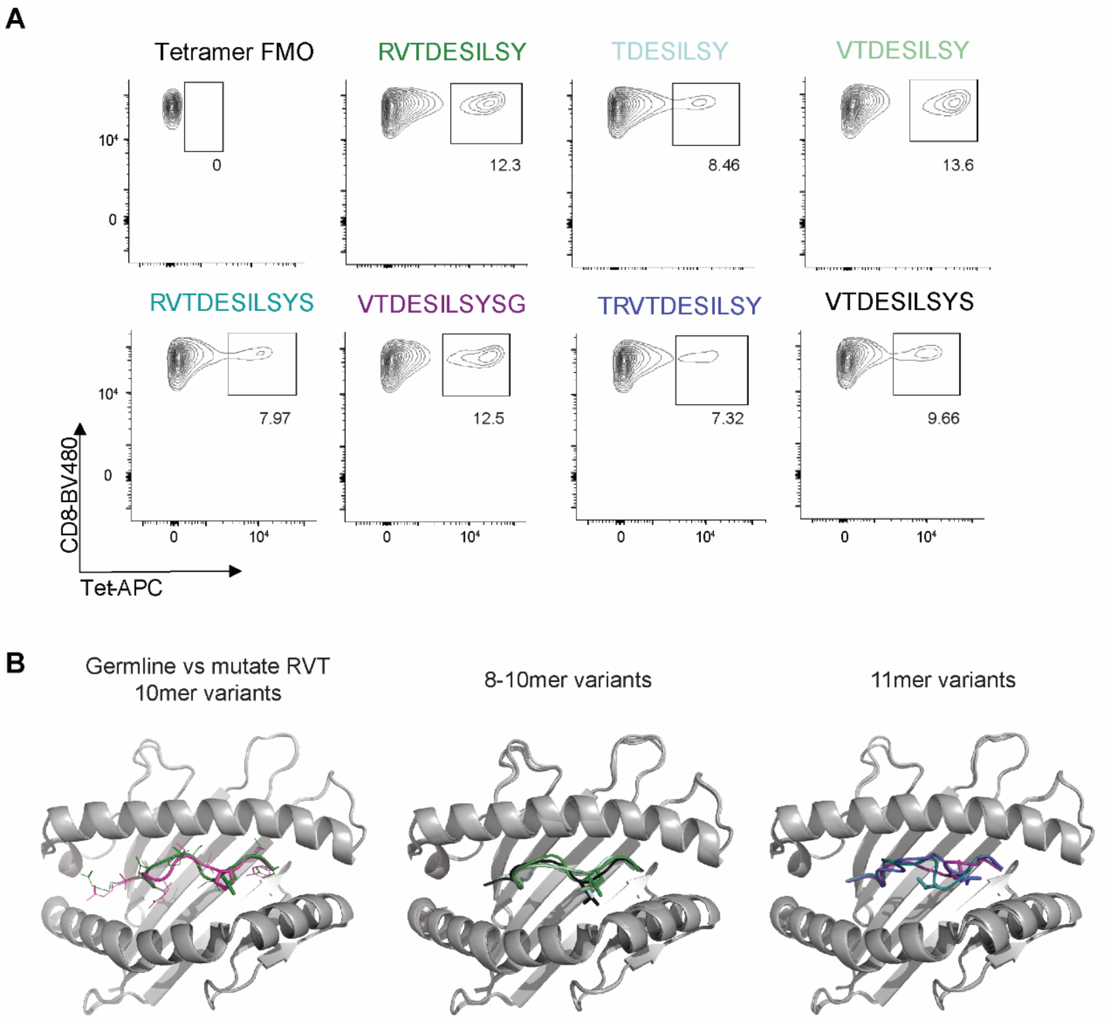

C

Mutated peptide	Germline peptide	Length	Est. freq (%)
RVTDES**L**SY	RVTDESPSY	10	9.88
TDES**L**SY	TDES**P**SY	8	2.06
VTDES**L**SY	VTDES**P**SY	9	1.92
RVTDES**L**SYS	RVTDESPSYS	11	1.10
VTDES**L**SYSG	VTDES**P**SYSG	11	0.91
VTDES**L**SYS	VTDES**P**SYS	10	0.47
TRVTDES**L**SY	TRVTDESPSY	11	0.36

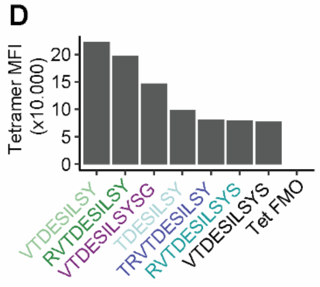

Supplemental Figure 3. Detection of NARTs specific for AKAP9^{P1796L} peptide variants ('SILSY' variants). (**A**) Dump channel negative (CD4-CD14-CD16-CD19-CD40-), neoepitope-specific CD8+ T cells from M22 TIL Inf product. All 7 predicted AKAP9^{P1796L} neopeptides tested with APC tetramers; all 7 were restricted to HLA-A*01:01. (**B**) Prediction of MHC binding confirmation to HLA-A*01:01 for all AKAP9^{P1796L} peptide variants using TCRpMHCmodels as described in the methods. (**C**) Summary table with peptide lengths and estimated frequencies of the NART population to each of the peptide variants. (**D**) MFI for the corresponding AKAP9^{P1796L} tetramer+ CD8 T cell populations in (A). MFI, median fluorescence intensity. Tet, tetramer. FMO, fluorescence minus one.

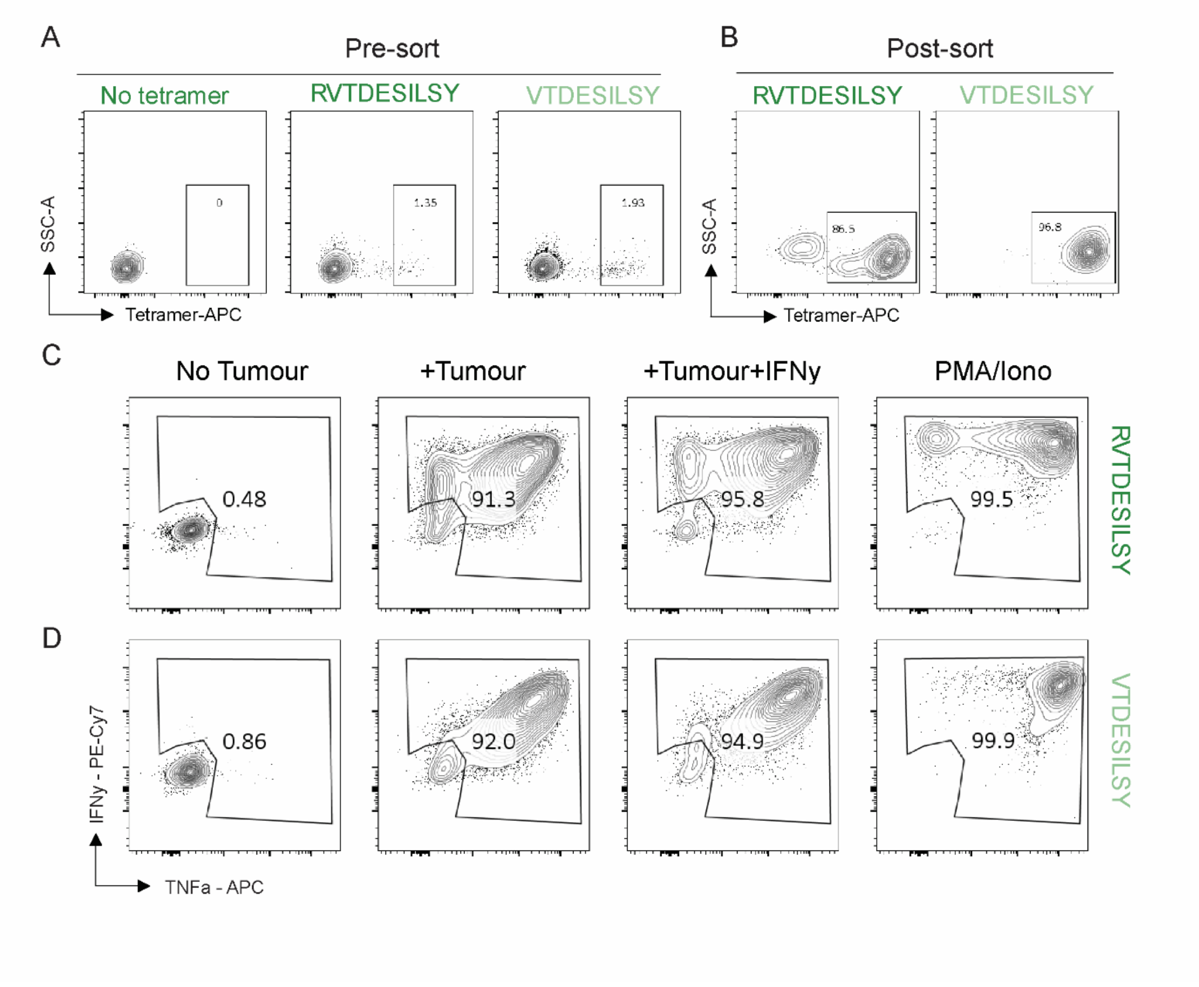

Supplemental Figure 4. Tumor recognition by expanded, AKAP9^{P1796L}-specifc NARTs. (**A**) HLA-A*01:01-restriced specific CD3+CD8+ T cells were sorted based on tetramer binding. (**B**) REP expanded cells were tested for neoepitope-recognition using tetramers. (**C-D)** IFNγ and TNFα release following co-cultures with autologous tumor cell lines and tetramer-specific clones recognizing RVTDESILSY (C) and VTEDILSY (D). All plots represent gated CD3+CD8+ T cells.

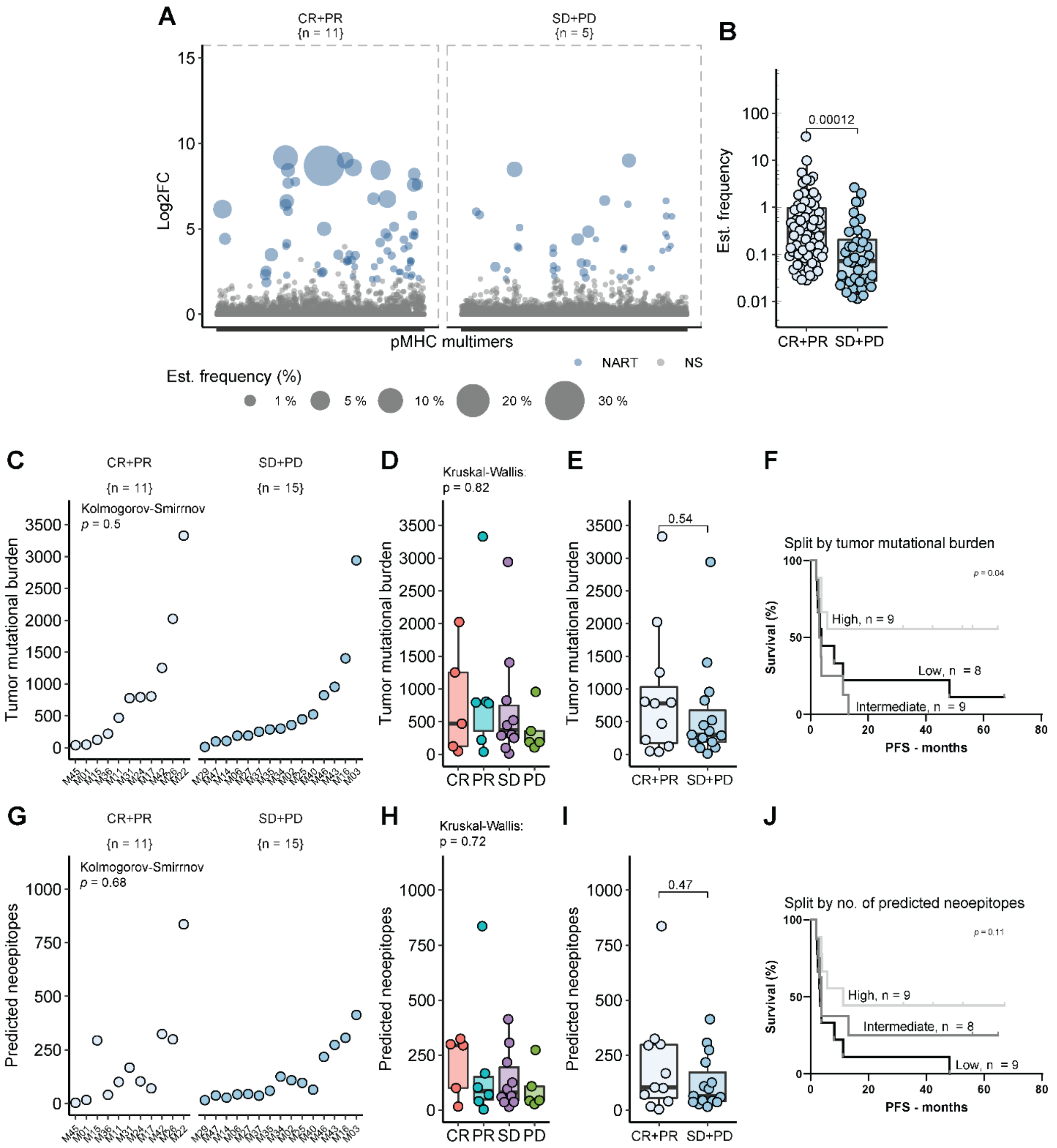
A
CR+PR
{n = 11}
SD+PD
{n = 5}
Log2FC
pMHC multimers
NART
NS
Est. frequency (%)
1 %
5 %
10 %
20 %
30 %
B
Est. frequency
0.00012
CR+PR
SD+PD
C
CR+PR
{n = 11}
SD+PD
{n = 15}
Kolmogorov-Smirnov
p = 0.5
Tumor mutational burden
D
Kruskal-Wallis:
p = 0.82
Tumor mutational burden
CR
PR
SD
PD
E
0.54
Tumor mutational burden
CR+PR
SD+PD
F
Split by tumor mutational burden
p = 0.04
High, n = 9
Low, n = 8
Intermediate, n = 9
Survival (%)
PFS - months
G
CR+PR
{n = 11}
SD+PD
{n = 15}
Kolmogorov-Smirnov
p = 0.68
Predicted neoepitopes
H
Kruskal-Wallis:
p = 0.72
Predicted neoepitopes
CR
PR
SD
PD
I
0.47
Predicted neoepitopes
CR+PR
SD+PD
J
Split by no. of predicted neoepitopes
p = 0.11
High, n = 9
Intermediate, n = 8
Low, n = 9
Survival (%)
PFS - months

Supplemental Figure 5. Tumor-mutational burden, estimated frequency and diversity of neoepitope-specific CD8 T cells in TIL-ACT. (**A**) All evaluated multimers and TIL Inf products plotted according to clinical response and the log2(fc) enrichment of the given barcode. Estimated frequency is depicted as the dot size. (**B**) Estimated frequency of all 106 NARTs detected in TIL Inf products. (**C-E**) Tumor mutational burden in the cohort; (**C**) according to patient sorted by highest tumor mutational burden; (**D**) according to RECIST; (**E**) Responders (CR+PR) vs non-responders (SD+PD). **(F)** Progression-free survival for the cohort split by tumor mutational burden. The 66th percentile = 787 mutations, 33rd percentile = 228.67 mutations. (**G-I**) Number of predicted neoepitopes; (**G**) according to patient sorted by highest tumor mutational burden; (**H**) according to RECIST; (**I**) Responders (CR+PR) vs non-responders (SD+PD). (**J)** Progression-free survival for the cohort split by number of predicted neoepitopes. The 66th percentile = 153 predicted neoepitopes, and the 33rd percentile = 49 predicted neoepitopes. Whiskers represent IQR. p-values were calculated using nonparametric Mann-Whitney U test in B, E and I. Kolmogorov-Smirnov was used in C and G to test equality of distributions. Kruskal-Wallis test was used for D and H. Finally, Log-rank and Mantel-Cox was used to calculate p-values and hazard ratios (HR) respectively for F and J.

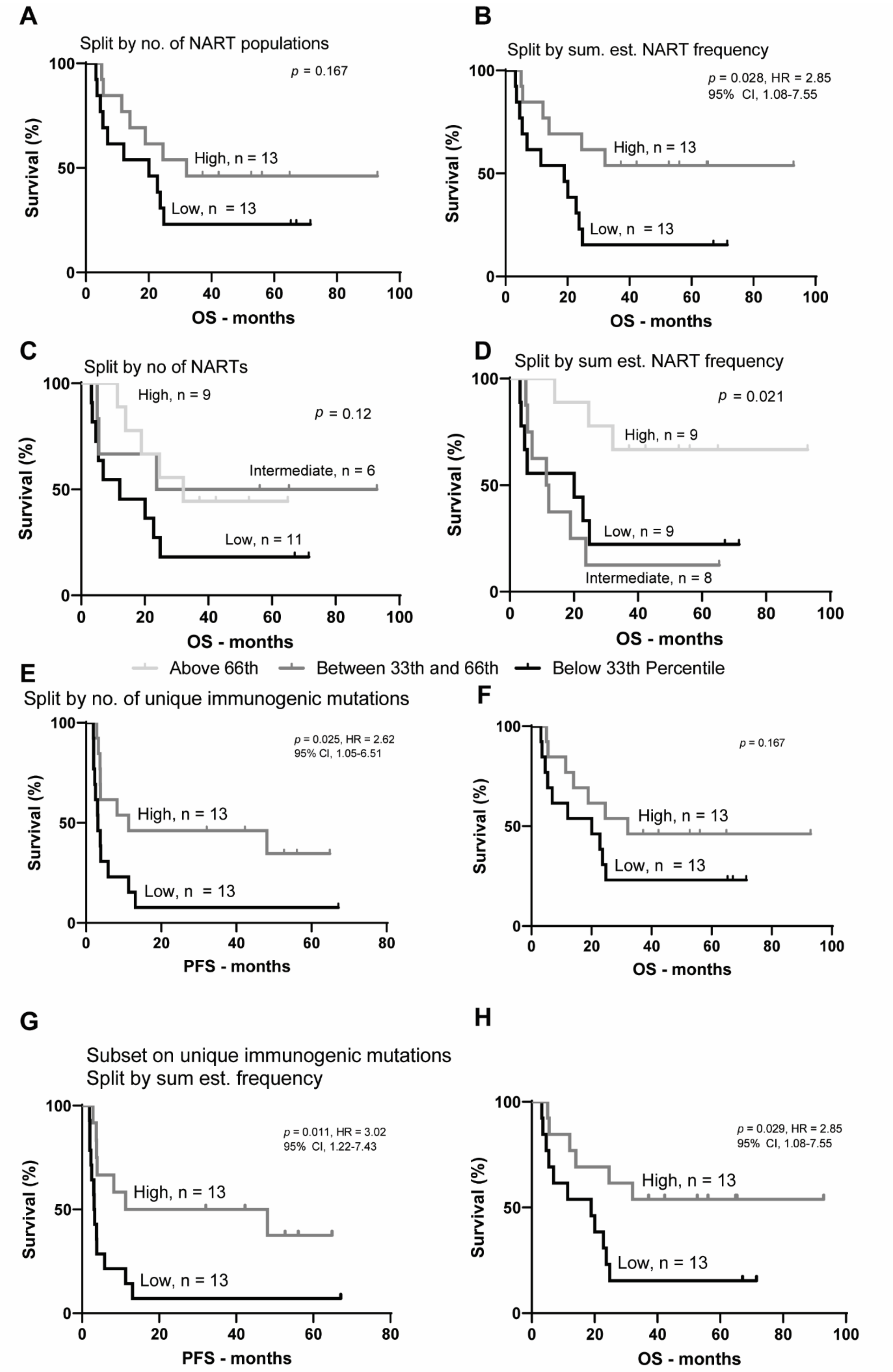
A
Split by no. of NART populations
p = 0.167
High, n = 13
Low, n = 13
Survival (%)
OS - months
B
Split by sum. est. NART frequency
p = 0.028, HR = 2.85
95% CI, 1.08-7.55
High, n = 13
Low, n = 13
Survival (%)
OS - months
C
Split by no of NARTs
High, n = 9
p = 0.12
Intermediate, n = 6
Low, n = 11
Survival (%)
OS - months
D
Split by sum est. NART frequency
p = 0.021
High, n = 9
Low, n = 9
Intermediate, n = 8
Survival (%)
OS - months
Above 66th
Between 33th and 66th
Below 33th Percentile
E
Split by no. of unique immunogenic mutations
p = 0.025, HR = 2.62
95% CI, 1.05-6.51
High, n = 13
Low, n = 13
Survival (%)
PFS - months
F
p = 0.167
High, n = 13
Low, n = 13
Survival (%)
OS - months
G
Subset on unique immunogenic mutations
Split by sum est. frequency
p = 0.011, HR = 3.02
95% CI, 1.22-7.43
High, n = 13
Low, n = 13
Survival (%)
PFS - months
H
p = 0.029, HR = 2.85
95% CI, 1.08-7.55
High, n = 13
Low, n = 13
Survival (%)
OS - months

127

Supplemental Figure 6. Impact of NART diversity and frequency, as well as presence of immunogenic mutations on overall and progression-free survival. (**A-B**), Overall survival split by median number of NARTs (3.65, A) or by median NART frequency (median = 0.64%, B) within TIL Inf product. (**C**) Overall survival split by 66th and 33rd percentile of NART diversity; 66th percentile = 5.65 NARTs. 33rd percentile = 0.88 NARTs. (**D**) Overall survival split by 66th and 33rd percentile of NART frequency; 66th percentile = 3.26%. 33rd percentile = 0.03%. (**E-F**), Overall **(E)** and Progression-free survival **(F)** split by median number of unique immunogenic mutations (3.22 uniquely recognized mutations). (**G-H**), Overall **(E)** and Progression-free survival **(F)** split by median NART frequency recognizing unique immunogenic mutations (0.63 %)**.** The highest estimated frequency among a group of NARTs recognizing the same mutation was taken as a proxy for the overall NART frequency of the group. p-values and hazard ratios (HR) from Mantel-Cox test and log-rank approach, respectively. Number of NARTs and NART frequency were normalized to HLA coverage as described in materials and methods. OS, Overall survival. PFS, Progression-free survival. n = 26 for all plots.

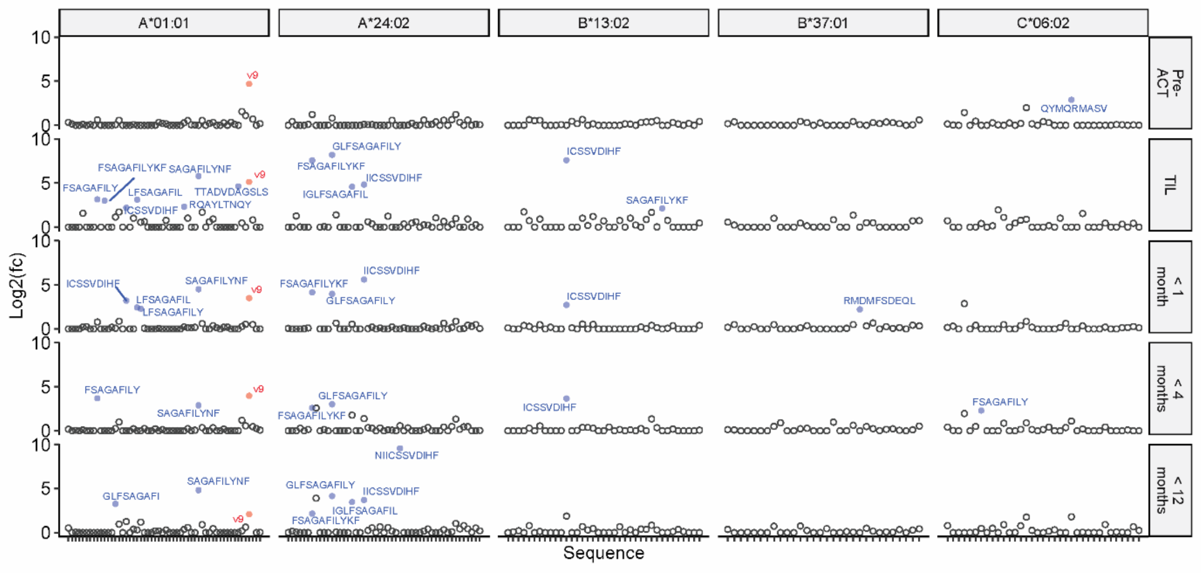

Supplemental Figure 7. NARTs in TIL samples and peripheral blood over time. Example of a full screen for CD8+ T cell populations in patient M45 (PR), in PBMC before and after therapy, and in the TIL Inf product. Separated according to HLA. Blue: NARTs. Red: responses to virus peptides. Grey dots were considered non-enriched barcodes. V9 annotates CMV peptide YSEHPTFTSQY.

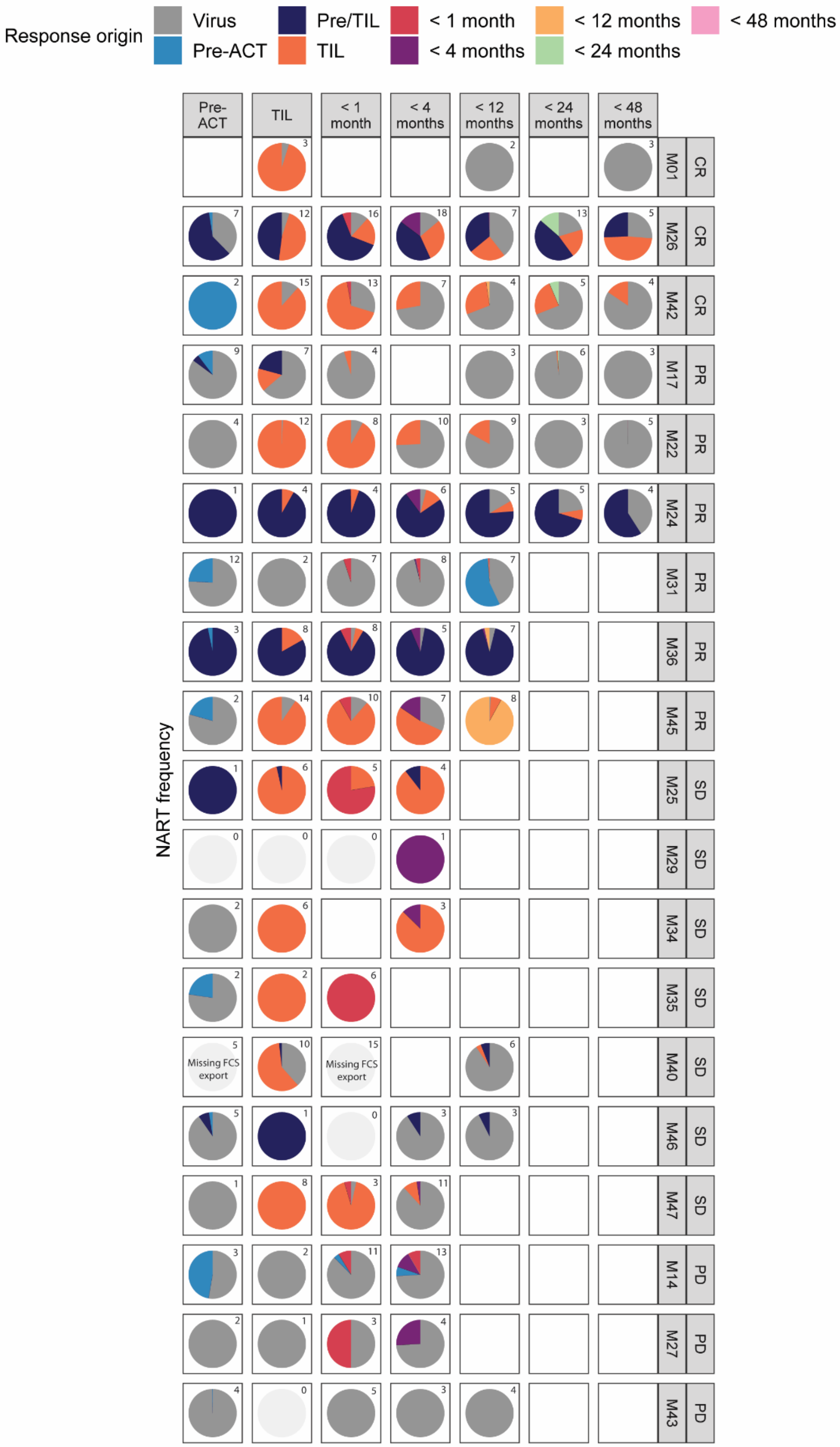

Response origin
Virus
Pre-ACT
Pre/TIL
TIL
< 1 month
< 4 months
< 12 months
< 24 months
< 48 months
Pre-ACT
TIL
< 1 month
< 4 months
< 12 months
< 24 months
< 48 months
NART frequency
M01 CR
M26 CR
M42 CR
M17 PR
M22 PR
M24 PR
M31 PR
M36 PR
M45 PR
M25 SD
M29 SD
M34 SD
M35 SD
M40 SD
M46 SD
M47 SD
M14 PD
M27 PD
M43 PD
Missing FCS export
Missing FCS export

Supplemental Figure 8. Distribution and temporal appearance of NARTs in TIL-ACT treated patients. Pie charts represent the frequency distribution of CD8+T cells specific towards neo- and viral epitopes followed over time from pre-ACT to < 48 months after therapy. Individual colors represents the group of NARTs appearing at a given time point. Virus responses are colored in grey. The total number of NART and virus responses within each circle is given in upper left corner for each time point. Missing FCS files from flow cytometry precludes frequency estimation in M40 Pre-ACT and <1 month PBMC samples.

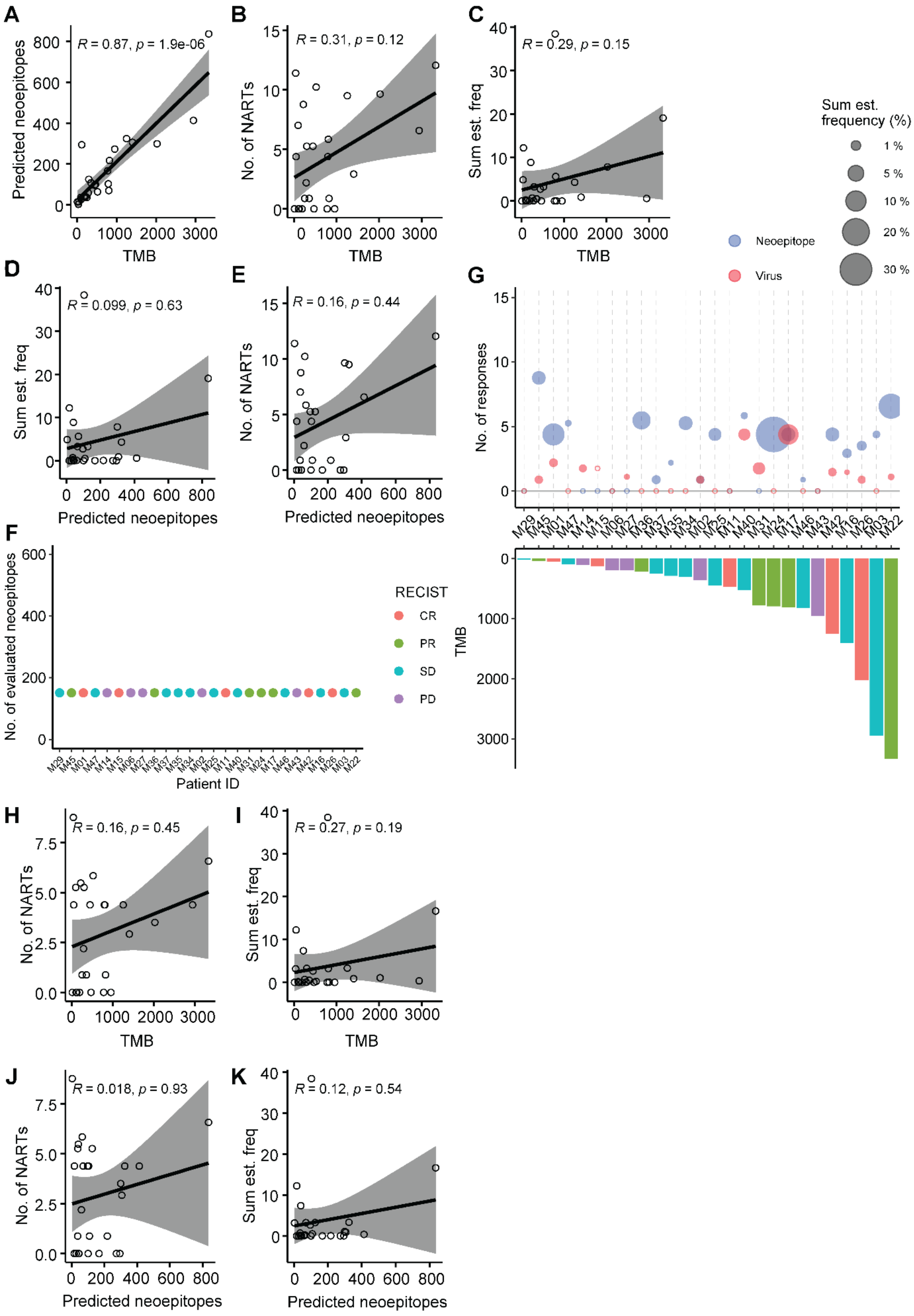

A
R = 0.87, p = 1.9e-06
Predicted neoepitopes
TMB
B
R = 0.31, p = 0.12
No. of NARTs
TMB
C
R = 0.29, p = 0.15
Sum est. freq
TMB
Sum est. frequency (%)
1 %
5 %
10 %
20 %
30 %
Neoepitope
Virus
D
R = 0.099, p = 0.63
Sum est. freq
Predicted neoepitopes
E
R = 0.16, p = 0.44
No. of NARTs
Predicted neoepitopes
G
No. of responses
M29 M45 M01 M47 M14 M15 M06 M27 M36 M37 M35 M34 M02 M25 M11 M40 M31 M24 M17 M46 M43 M42 M16 M26 M03 M22
TMB
F
No. of evaluated neoepitopes
RECIST
CR
PR
SD
PD
Patient ID
H
R = 0.16, p = 0.45
No. of NARTs
TMB
I
R = 0.27, p = 0.19
Sum est. freq
TMB
J
R = 0.018, p = 0.93
No. of NARTs
Predicted neoepitopes
K
R = 0.12, p = 0.54
Sum est. freq
Predicted neoepitopes

Supplemental Figure 9. NART diversity and frequency within TIL Inf products does not correlate with tumor mutational burden or number of predicted neoepitopes. (**A**) TMB vs. number of predicted neoepitopes. **(B-E)** All evaluated multimers (151-585 multimers per patient). (**B**) TMB vs NART diversity. (**C**) TMB vs NART frequency. (**D**) Number of predicted neoepitopes vs NART diversity. (**E**) Number of predicted neoepitopes vs NART frequency. (**F**) Alternative selection strategy used for G through K selecting top 151 neoepitopes with the highest binding potential according to predicted %rank score. (**G**) NART diversity and frequency following alternative selection. Patients were arranged according to TMB. (**H**) TMB vs NART diversity. (**I**) TMB vs NART frequency. (**J**) Number of predicted neoepitopes vs NART diversity. (**K**) Number of predicted neoepitopes vs NART frequency. Diversity and frequency values were normalized to HLA coverage (see materials and methods). R and p-values from Spearman correlation with 95% confidence intervals in grey. All patients were evaluated (n = 26).

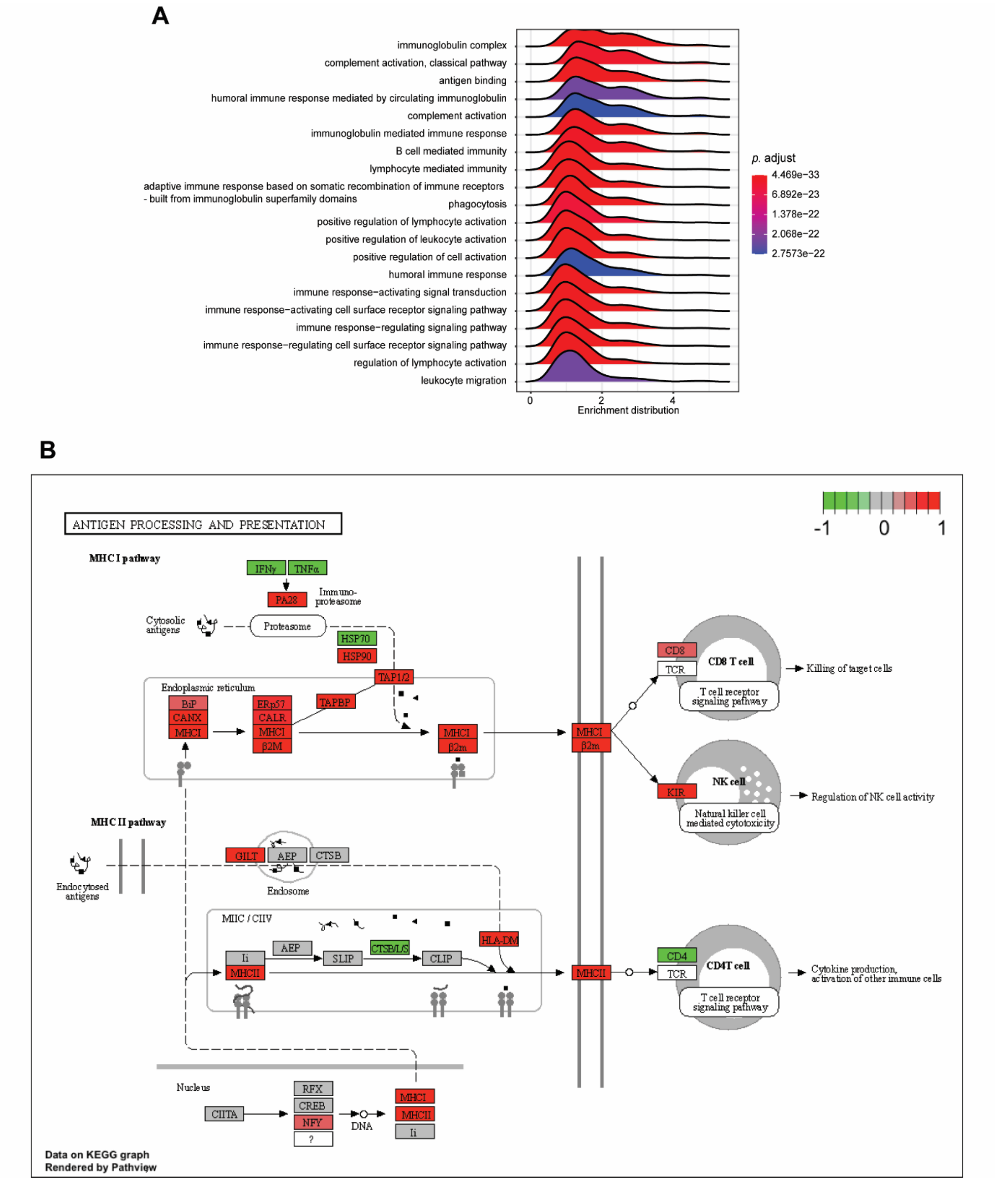

Supplemental Figure 10. Exploratory analysis of differentially expressed genes. (**A**) Top 20 enriched gene sets according to GO terms. (**B**) KEGG-pathway analysis showing the Antigen processing and presentations pathway colered by enriched genes according to the GSEA for enriched GO terms. Significance threshold were set with an FDR $\leq$ 0.01.
